# Causal relationship between gut microbiota and thyroid nodules: a bidirectional two-sample Mendelian randomization study

**DOI:** 10.3389/fendo.2024.1417009

**Published:** 2024-08-08

**Authors:** Shaoshuai Yan, Jiawei He, Xudong Yu, Jianwei Shang, Yaosheng Zhang, Han Bai, Xingyu Zhu, Xiaoming Xie, Leanne Lee

**Affiliations:** ^1^ Dongzhimen Hospital, Beijing University of Chinese Medicine, Beijing, China; ^2^ School of Traditional Chinese Medicine, Beijing University of Chinese Medicine, Beijing, China; ^3^ School of Traditional Chinese Medicine, Hubei University of Chinese Medicine, Wuhan, Hubei, China

**Keywords:** gut microbiota, thyroid nodules, Mendelian randomization, causal association, thyroid-gut axis

## Abstract

**Objective:**

Emerging evidence suggests alterations in gut microbiota (GM) composition following thyroid nodules (TNs) development, yet the causal relationship remains unclear. Utilizing Mendelian Randomization (MR), this study aims to elucidate the causal dynamics between GM and TNs.

**Methods:**

Employing summary statistics from the MiBioGen consortium (*n*=18,340) and FinnGen consortium (1,634 TNs cases, 263,704 controls), we conducted univariable and multivariable MR analyses to explore the GM-TNs association. Techniques including inverse variance weighted, MR-Egger regression, weighted median, and MR-PRESSO were utilized for causal inference. Instrumental variable heterogeneity was assessed through Cochran’s *Q* statistic and leave-one-out analysis. Reverse MR was applied for taxa showing significant forward MR associations, with multivariate adjustments for confounders.

**Results:**

Our findings suggest that certain microbiota, identified as Ruminococcaceae_NK4A214_group (OR, 1.89; 95%CI, 0.47-7.64; p = 0.040), Senegalimassilia (OR, 1.72; 95%CI, 1.03-2.87; p =0.037), Lachnospiraceae (OR,0.64; 95%CI,0.41-0.99; p =0.045), exhibit a protective influence against TNs’ development, indicated by negative causal associations. In contrast, microbiota categorized as Desulfovibrionales (OR, 0.63; 95%CI, 0.41-0.95; p =0.028), Prevotella_7 (OR, 0.79; 95%CI, 0.63-1.00; p =0.049), Faecalibacterium (OR, 0.66; 95%CI, 0.44-1.00; p =0.050), Desulfovibrionaceae (OR, 0.55; 95%CI, 0.35-0.86; p =0.008), Deltaproteobacteria (OR, 0.65; 95%CI, 0.43-0.97; p =0.036) are have a positive correlation with with TNs, suggesting they may serve as risk factors. Reverse MR analyses did not establish significant causal links. After comprehensive adjustment for confounders, taxa Desulfovibrionales (Order), Desulfovibrionaceae (Family), Deltaproteobacteria (Class) remain implicated as potential contributors to TNs’ risk.

**Discussion:**

This study substantiates a significant causal link between GM composition and TNs development, underscoring the thyroid-gut axis’s relevance. The findings advocate for the integration of GM profiles in TNs’ prevention and management, offering a foundation for future research in this domain.

## 1 Introduction

Thyroid nodules (TNs), considered localized enlargements of the thyroid gland, represent a prevalent category of endocrine disorders. The annual incidence of TNs is escalating, with the prevalence in females being approximately triple that observed in males (1, 2). During long-term follow-up, an increase in nodule grading is observed in a subset of patients with TNs. Among these patients, the proportion diagnosed with thyroid cancer (TC) via fine-needle aspiration biopsy ranges from 4.0% to 6.5% (3). TNs poses a substantial burden on global health (4) and exerts significant psychological stress on patients. Research has identified several risk factors for TNs, including exposure to radiation, genetic predispositions, estrogen levels, Body Mass Index (BMI), smoking habits, and metabolic syndrome (5, 6). Recently, the correlation between the gut microbiota (GM) and thyroid health has attracted considerable attention. However, the specific pathogenesis of TNs remains unclear, and effective clinical treatment strategies are lacking.

The GM, also known as gut flora, comprises a complex ecosystem of bacteria, viruses, fungi, and other microorganisms inhabiting the human gastrointestinal tract. This ecosystem, predominantly bacterial in healthy adults, includes a mix of anaerobic and aerobic organisms, with a notable presence of bifidobacteria and lactobacilli (7). The GM plays an essential role in health, supporting digestion, nutrient absorption, metabolism, and immune function. However, alterations in diet, medication use, and stress can disrupt the microbial balance, potentially favoring pathogenic bacteria and triggering or exacerbating diseases (8).

In the past decade, a large number of studies have been devoted to exploring the impact of microbial communities on various diseases, affirming their critical role. A causal link has been established between GM disturbances and diseases affecting the nervous, cardiovascular, and respiratory systems, among others (9–11), including disorders of the thyroid, a key organ within the endocrine system. The thyroid and gastrointestinal epithelium, both of endodermal origin, exhibit functional and morphological similarities that contribute to mutual regulation, maintaining both thyroid and gastrointestinal homeostasis. The high co-occurrence of thyroid and gastrointestinal diseases underlines a potential thyroid-gut axis (12). Thyroid hormones are known to enhance gastrointestinal motility, with thyroid dysfunction often manifesting as gastrointestinal complaints such as dyspepsia, diarrhea, constipation, and microbiota dysbiosis (13). The GM influences various thyroid conditions, including Hashimoto’s thyroiditis, TC, hyperthyroidism, and hypothyroidism (14–17). The GM possesses a variety of degradative enzymes that are involved in the metabolism of various nutrients, including iodine, selenium, zinc, and iron. It also plays a crucial role in the synthesis and regulation of minerals and vitamins. The mechanisms by which the GM affects the thyroid may involve influencing deiodinase activity and inhibiting TSH, directly impacting thyroid hormone levels and potentially leading to thyroid dysfunction (18, 19). Additionally, the GM may promote inflammation and reduce immune tolerance, thereby altering immune responses (20). This can disrupt the intestinal mechanical and immune barriers, increase intestinal permeability, promote bacterial translocation, and ultimately trigger local and systemic inflammatory responses. Studies have linked GM composition and its metabolic activities, such as butyrate production, to the presence of TNs and their TI-RADS grading (21), suggesting a reciprocal impact on thyroid function (22). Early identification of TN risk factors is imperative for the development of diagnostic and therapeutic strategies. Currently, the primary methods for intervening in GM include the administration of probiotics and fecal microbiota transplantation.

Mendelian Randomization (MR), using Single Nucleotide Polymorphisms (SNPs) as instrumental variables (IVs) for genome-wide association studies (GWAS), is utilized to infer causal relationships between exposures and outcomes (23). Emulating the random allocation mechanism of randomized controlled trials (RCTs) through the distribution of alleles at conception, MR offers a powerful tool to circumvent the limitations and biases inherent in observational studies (24). The application of MR in delineating causal pathways among complex diseases has been extended to explore the connections between GM and a range of conditions, including autoimmune, cardiovascular, and psychiatric disorders.

However, the causal relationship between GM and TNs remains undefined. As a critical environmental factor in disease pathogenesis, GM warrants focused investigation. Systematic reviews indicate a lack of studies on the causal association between GM and TNs. Therefore, this study aims to employ MR analysis, utilizing GWAS summary data from the MiBioGen and FinnGen consortia, to elucidate the bidirectional causal relationship between GM and TNs. This research aspires to provide novel insights for the prevention and management of TNs.

## 2 Materials and methods

### 2.1 Ethics statement and study design

This investigation utilizes publicly available data of international acclaim, compliant with the Declaration of Helsinki and sanctioned by pertinent ethical bodies (the MiBioGen Consortium and the FinnGen research consortium). Such foundational datasets have garnered ethics and institutional review board endorsements, obviating the necessity for further ethical scrutiny. Selection of SNPs significantly linked with exposures as IVs was executed adhering to rigorous selection paradigms, incorporating these IVs into MR and sensitivity analyses based on the hypothetical constructs of MR.

**Figure 1:**
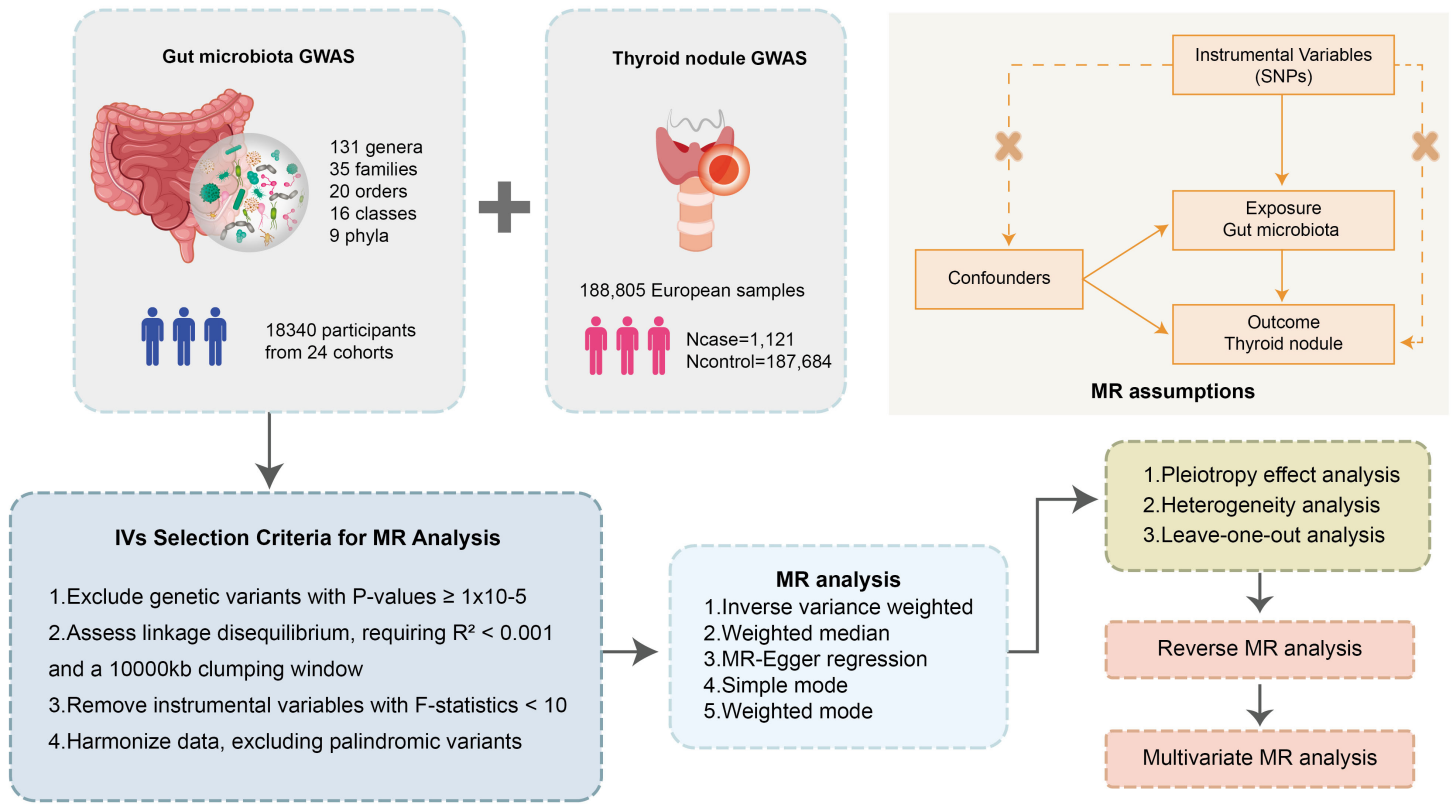
Flowchart of the MR randomization study and key assumptions. MR, Mendelian randomization; SNPs, single nucleotide polymorphisms; IVW, inverse-variance weighted; MR-PRESSO, MR pleiotropy residual sum and outlier.

The focal point of this research is the examination of the GM and TNs as exposures to discern their implications on specific outcomes. The MR analytical framework employed herein is anchored in three cardinal assumptions (25). Firstly, it is verified that the genetic variations possess independent and significant correlations with the exposures, with the instrumental variable SNPs demonstrating substantial linkage to GM and TNs, as ascertained through *F*-statistics and *p*-values within correlation analyses. In the second place, it is validated that the genetic variations pertinent to the exposures are not directly correlated with the outcomes, with SNPs influencing TNs solely via GM. Lastly, it is presupposed that these variables are exempt from any association with confounding factors (**Figure 1**).

The study’s reporting of this study is in strict alignment with adheres closely to the Guidelines on Strengthening the Reporting of Observational Studies in Epidemiology using Mendelian Randomization (STROBE-MR) (26), guaranteeing thorough and transparent disclosure of methodological and analytical details (**Supplementary Table S1**).

### 2.2 Data sources

The data on the human GM is derived from the largest GWAS published to date, representing the most extensive multi-ethnic, whole-genome analysis of human autosomal genetic variations and the GM (27). The research includes 18,340 participants from various nations/areas, primarily representing the European population. The research successfully identified 211 bacterial taxa, including 9 phyla, 16 classes, 20 orders, 35 families (3 of which are unknown), and 131 genera (12 unknown). After excluding 15 taxa lacking family and genus information, the study conducted a preliminary analysis on the remaining 196 bacteria (details shown in **Supplementary Table S2**).

Moreover, summary data for TNs’ GWAS were obtained from the FinnGen consortium, which includes 1,634 cases and 263,704 controls, all of European descent (details shown in **Supplementary Table S2**).

Summary data for GWAS on risk factors for TNs (including high blood pressure, obesity class 3, alcohol consumption, type 2 diabetes, and ever smoked) were sourced from the corresponding consortium within the Integrative Epidemiology Unit (IEU) Open GWAS project (**Supplementary Table S2**).

### 2.3 Selection of instrumental variables

In this investigation, SNPs manifesting a *p*-value below 1×10^-5^ were harnessed as preliminary genetic instrumental variables for the MR analysis. This *p*-value threshold has been identified as optimally conducive across numerous MR studies investigating the GM, aiming to enhance the genetic variance explained by genetic predictors and to expand the array of SNPs available for sensitivity analysis (28). Furthermore, the *F*-statistic is employed as a robust measure to access the stability of the instrumental variables.


$$F=\frac{R^{2}(n-1-k)}{\left(1-R^{2}\right)k}$$

It is typically derived using a formula where *R^2^* denotes the variance elucidated by the instrumental variable, *n* symbolizes the sample size, and *k* denotes the number of selected IVs. The *F*-statistic is calculated to ascertain the strength of the chosen instrumental variables, with an *F*-value exceeding 10 signaling that the instruments are robust against the weak instrument bias (29); SNPs exhibiting an *F*< 10 were disqualified.

To mitigate the impact of linkage disequilibrium (LD) on the results, the included genetic variants were clustered using an *R^2^* threshold greater than 0.001 and a clustering window of less than 10,000 kb (30), to sift through the genetic variations. Subsequent to this, SNPs associated with TNs were disqualified based on the criterion: *P*_outcome_< *P*_exposure_. Lastly, SNPs featuring palindromic structures were eliminated to guarantee that the filtered SNPs were congruently oriented in terms of alleles for both exposure and outcome, thus refining the integrity of the analytical framework.

### 2.4 Statistical analysis of mendelian randomization

For the univariate Mendelian randomization (UVMR) analysis, the inverse variance weighted (IVW) method, the main tool for exploring the relationship between GM and TNs. This approach synthesizes Wald estimators across SNPs to deduce the causal impact of exposure on the result, amalgamating ratio estimates from each genetic variant (31), and provides the most precise estimations when the genetic variants are valid IVs (32). Additionally, adjunctive analytical techniques such as MR-Egger regression, weighted median, simple mode, and weighted mode are utilized.

Primarily, MR-Egger regression addresses pleiotropy concerns (33, 34), with significance in the MR-PRESSO (*p*< 0.05) bolstering the validity of positive outcomes. A suite of sensitivity analyses ensures the robustness of results. Cochran’s *Q* test within the IVW framework and via MR-Egger regression (considering *p*< 0.05 as indicative of potential heterogeneity) assesses IV heterogeneity. Leave-one-out sensitivity analysis evaluates outliers and the stability of results (33). MR-Egger and MR-PRESSO tests identify pleiotropy and outliers, with the MR-Egger intercept specifically testing for horizontal pleiotropy. The absence of significant horizontal pleiotropy is suggested by *p* > 0.05. MR-PRESSO, compared to MR-Egger, more accurately detects horizontal pleiotropy and outliers (35).

To fully understand the connection between GM and TNs, reverse MR analysis was also performed on GM causally associated to TNs in the forward MR assessment, using the same settings and methodologies. Moreover, potential confounders such as alcohol consumption, high blood pressure, obesity class 3, smoking status, and type 2 diabetes could influence the causal link between GM and TNs. To account for these confounders, the study incorporated these factors into the forward MR analysis via multivariable Mendelian randomization (MVMR). IVW, MR-Egger regression, and weighted median methods were applied in MVMR analyses, with Cochran’s *Q* tests evaluating the consistency of outcomes.

Statistical analyses were performed using R software (version 4.3.1), employing the TwoSampleMR, MR-PRESSO, and Mendelian Randomization R packages for MR investigations.

## 3 Results

### 3.1 Selection of instrumental variables

In this study, adhering to the instrumental variable selection criteria with an *F*-statistic greater than 10 to mitigate the risk of weak instrumental bias, 196 GM were associated with 2, 077 SNPs in total. Among these, 102 SNPs were related to 9 phyla, 178 SNPs to 16 classes, 216 SNPs to 20 orders, 351 SNPs to 32 families, and 1, 230 SNPs to 119 genera. The analysis utilized the IVW method as the primary reference, with *p*< 0.05 as the threshold, to conduct MR analysis on 196 bacterial taxa. A total of 163 bacterial groups from 16 groups were found to be associated with TNs. These groups include 1 class (14 SNPs), 1 order (13 SNPs), 2 families (29 SNPs), and 5 genera (60 SNPs) (**Supplementary Table S3**).

### 3.2 Causal effects of gut microbiota on thyroid nodules by UVMR

**Figure 2:**
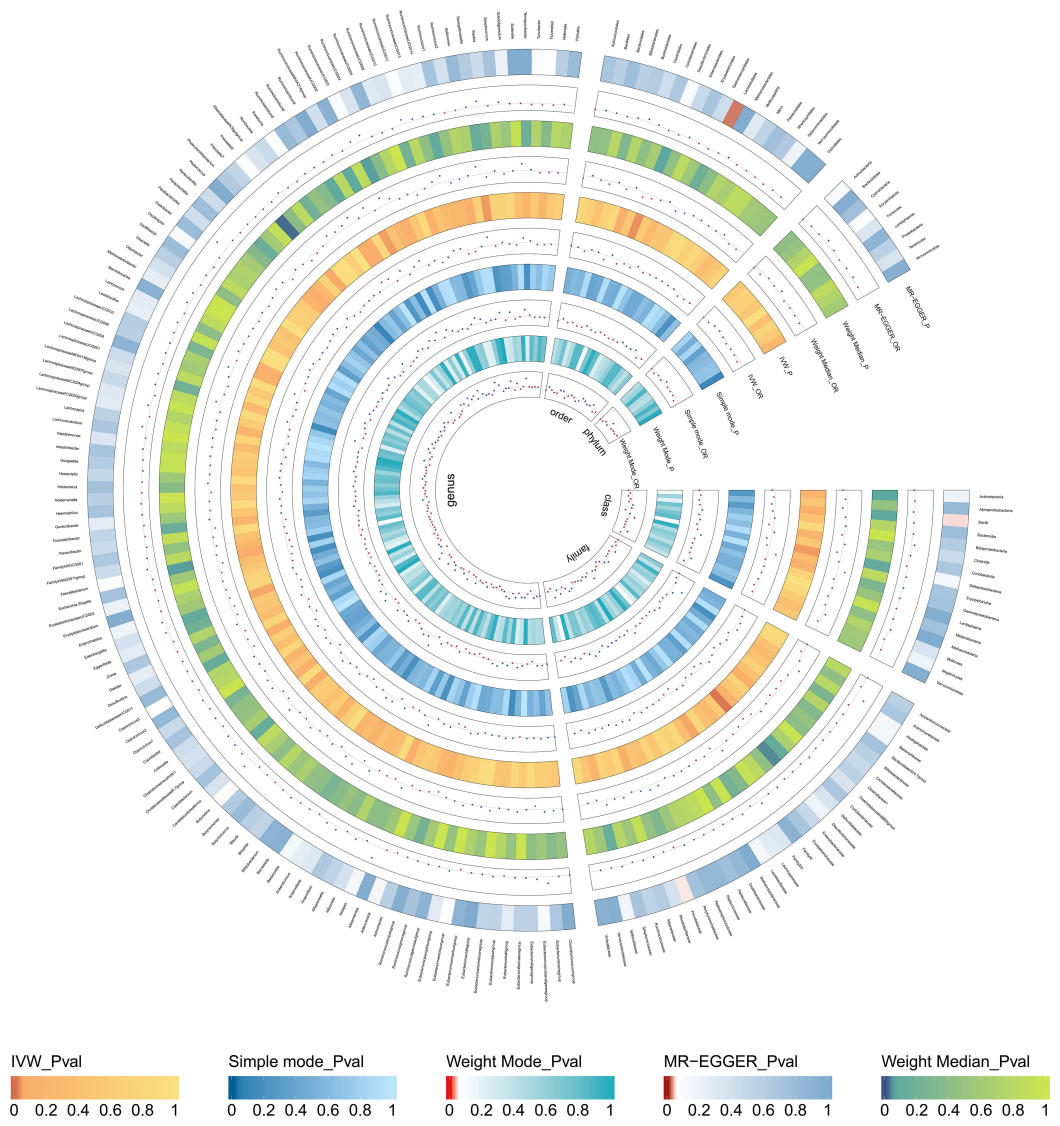
Causal effect of the gut microbiota on thyroid nodules based on MR analyses. From outside to inside, the p-values and OR of MR-Egger, weighted median, inverse variance weighted(IVW), simple mode, and weighted mode are represented, respectively.

**Figure 3:**
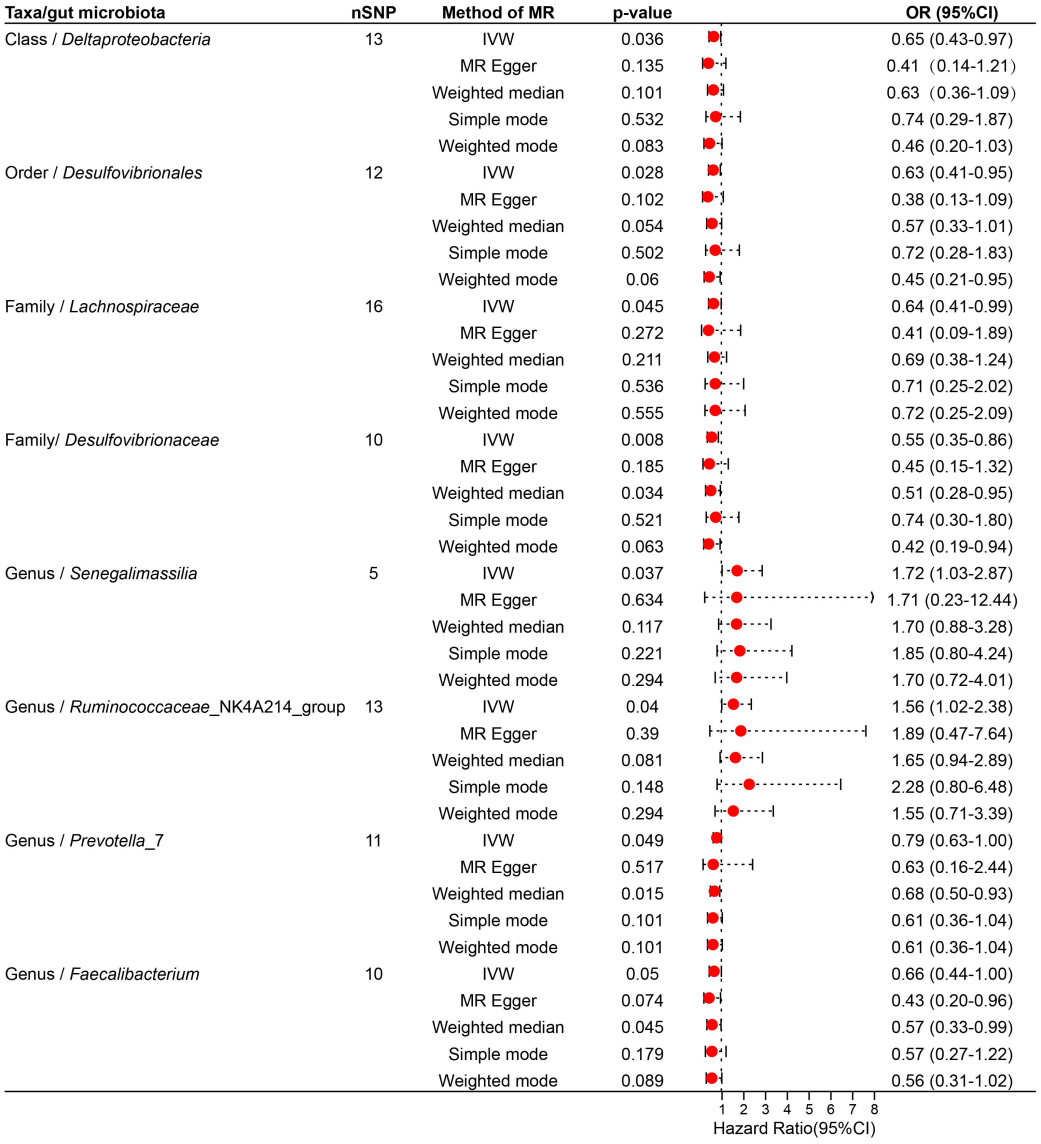
Forest plot illustrating the causal effect of the gut microbiota on thyroid nodules using five methods of MR analyses.

The results of MR Analysis are shown in **Figure 2**, where the beta values for all MR analysis methods and the *p*-values for IVW methods are recorded. Positive results from the MR analysis were identified based on significant *p*-values from the IVW. The IVW revealed that 9 taxa of GM are related to TNs. However, the beta results from the five methods for *Eisenbergiella* (Genus) showed inconsistent directions, and to ensure the study outcomes were as free from confounding factors and horizontal pleiotropy as possible, they were excluded. The direction of the beta results from all five methods for the remaining taxa was consistent, enhancing the credibility of a true causal relationship (**Supplementary Table S4**; **Figure 3**). The *F*-statistics of the IVs included in the analysis that were significantly associated with the GM were all greater than 10, suggesting that the estimates are less susceptible to weak instrumental bias (**Supplementary Table S1**).

Specifically, *Ruminococcaceae*_NK4A214_group (Genus) (*OR*, 1.89; 95%CI, 0.47-7.64; *p* = 0.040), *Senegalimassilia* (Genus) (*OR*, 1.72; 95%CI, 1.03-2.87; *p* =0.037), *Lachnospiraceae* (Family) (*OR*,0.64; 95%CI,0.41-0.99; *p* =0.045) presented a negative causal association with TNs, potentially acting as a protective factor against the onset of TNs. Conversely, an increase in the abundance of *Desulfovibrionales* (Order) (*OR*, 0.63; 95%CI, 0.41-0.95; *p* =0.028), *Prevotella*_7 (Genus) (*OR*, 0.79; 95%CI, 0.63-1.00; *p* =0.049), *Faecalibacterium* (Genus) (*OR*, 0.66; 95%CI, 0.44-1.00; *p* =0.050), *Desulfovibrionaceae* (Family) (*OR*, 0.55; 95%CI, 0.35-0.86; *p* =0.008), *Deltaproteobacteria* (Class) (*OR*, 0.65; 95%CI, 0.43-0.97; *p* =0.036) may induce the occurrence of TNs, with higher abundance of these taxa associated with an increased potential risk of TNs (**Supplementary Table S4**; **Figure 3**).

### 3.3 Sensitivity analysis

We performed pleiotropy, heterogeneity test, and leave-one-out analysis in sensitivity analyses. The regression intercepts of all MR-Egger regressions were not significantly deviated from zero (all intercepts *p* > 0.05). Cochran’s *Q* test and MR-PRESSO *p*-values were greater than 0.05; all of these conditions indicated the absence of significant pleiotropy and heterogeneity (as shown in **Supplementary Tables S5**, **S6**). In addition, leave-one-out analysis revealed no specific SNP driving the link between GM and TNs (as shown in **Supplementary Table S7**).

### 3.4 Causal effects of thyroid nodules on gut microbiota

Further, a reverse MR analysis was specifically conducted on GM with positive results to explore whether there is a causal relationship between TNs and GM. With TNs as the exposure and the GM (*Ruminococcaceae*_NK4A214_group, *Senegalimassilia*, *Lachnospiraceae*, *Desulfovibrionales, Prevotella*_7, *Faecalibacterium*, *Desulfovibrionaceae*, *Deltaproteobacteria*) with a definitive causal relationship with bacterial pneumonia as the outcome, reverse MR was conducted (shown in **Supplementary Tables S8**, **S9**). No heterogeneity or pleiotropy was found in the reverse MR analysis, and leave-one-out analysis indicated that no SNP were significantly linked with the outcome (**Supplementary Tables S10**, **S11**).

A total of 40 instrumental variables were included under the screening conditions of *p*<1×10^-5^, *r^2^ =* 0.001, and kb=10,000, but no significant reverse causal relationships were detected in subsequent MR analyses.

### 3.5 Causal effects of thyroid nodules on gut microbiota

**Figure 4:**
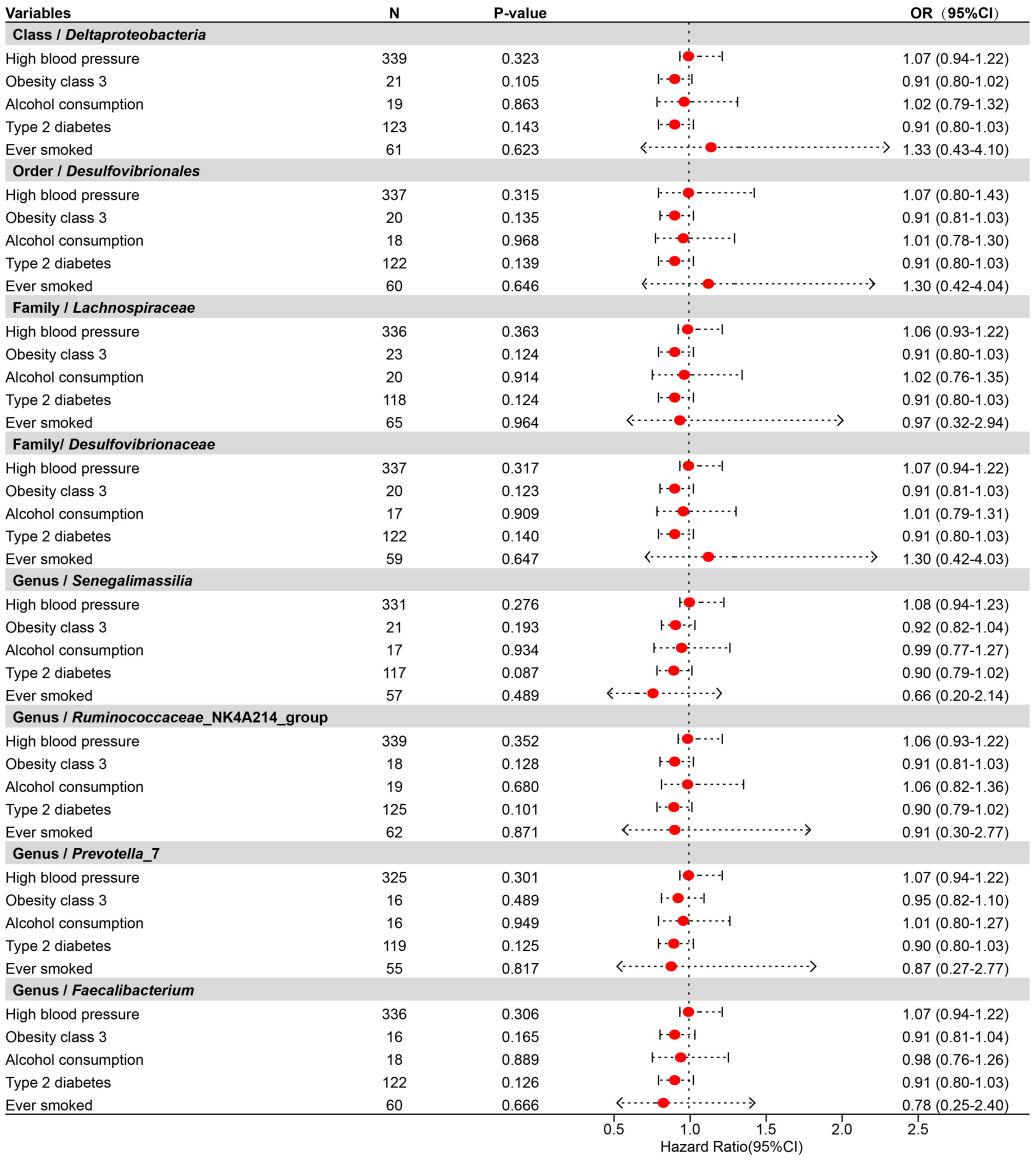
Forest plot illustrating the causal effect of gut microbiota on thyroid nodules using the IVW method to adjust for each of the five confounders (including high blood pressure, obesity class 3, alcohol consumption, type 2 diabetes, and ever smoked).

**Figure 5:**
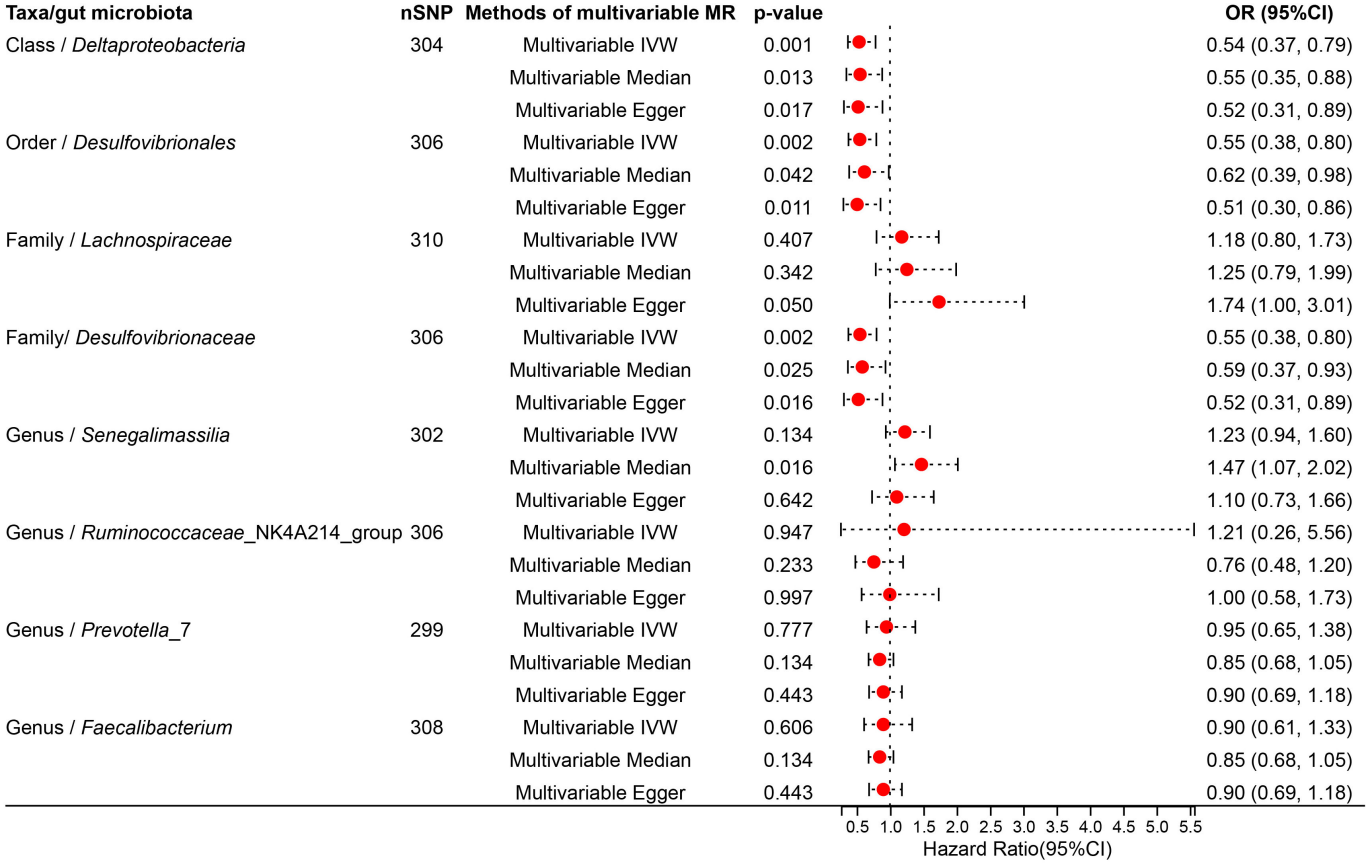
Forest plot illustrating the causal effect of gut microbiota on thyroid nodules using IVW methods with simultaneous adjustment for five confounders.

To determine whether the aforementioned microbiota directly or indirectly impact the risk of TNs through common confounding factors, further MVMR analyses were performed. In MR analyses with TNs as the outcome, confounders such as high blood pressure, obesity class 3, alcohol consumption, type 2 diabetes, and ever smoked, were considered, with the final results shown in **Figure 4**, **Supplementary Table S12**. After simultaneously considering all confounders, the results were shown in **Figure 5**, **Supplementary Table S13**. To be specific, *Desulfovibrionales* (Order), *Desulfovibrionaceae* (Family), *Deltaproteobacteria* (Class) remained a potential risk factor for TNs when TNs was the outcome. The consistent direction across different MR models further supports the causal inference. Moreover, Cochran’s *Q* from multivariable IVW and multivariable MR-Egger tests and intercepts detected no potential pleiotropy and heterogeneity (**Supplementary Table S14**).

## 4 Discussion

In the context of escalating global health consciousness, the detection rates of TNs have experienced a marked increase, metaphorically likened to a “time bomb” profoundly impacting patient emotional well-being. Clinically, individuals diagnosed with TNs frequently exhibit symptoms of depression and anxiety, conditions that are closely linked with gastrointestinal disorders. Recent epidemiological studies have noted a significant escalation in TNs prevalence among individuals with persistent Helicobacter pylori infection (36). The pathogenesis of TNs remains complex and multifaceted, influenced by environmental, genetic, and autoimmune factors, underscoring the critical need for further research and exploration. Although a substantial body of evidence underscores the association between thyroid pathologies and the composition of the GM, research on the role of GM in patients with TNs remains scant. This investigation delves into the bidirectional causal relationship between the GM and TNs, providing new perspectives.

This study reveals a negative causal association between GM, considered as an exposure factor, and TNs as an outcome, identifying specific microbial taxa (*Ruminococcaceae*_NK4A214_group, *Senegalimassilia*, *Lachnospiraceae*) as potential protective factors against TNs development. In contrast, other microbial taxa (*Desulfovibrionales*, *Prevotella*_7, *Faecalibacterium*, *Desulfovibrionaceae*, *Deltaproteobacteria*) exhibit a positive causal relationship with TNs, potentially serving as biomarkers of the disease. Upon adjusting for confounding variables, certain taxonomic classifications—*Desulfovibrionales* (Order), *Desulfovibrionaceae* (Family), *Deltaproteobacteria* (Class)—persist as potential risk factors for TNs development.

The *Ruminococcaceae*_NK4A214_group, a member of the *Ruminococcaceae* family, is related in the gut’s energy metabolism and fatty acid synthesis, particularly in the fermentation of complex carbohydrates to produce butyrate, noted for its anti-inflammatory properties (37). The association between butyrate production and thyroid disorders, including TNs, is well established. A clinical study by Ang Li et al. involving 196 TNs patients and a cohort of healthy controls demonstrated a reduction in the diversity and number of gene families within the GM of TNs patients, alongside a decreased relative abundance of butyrate-producing microbes, a finding especially pronounced in patients with advanced TNs (21). The diminished expression of NIS mRNA characterizes benign nodules, suggesting an epigenetic pattern intermediate between normal thyroid tissue and malignant TNs (38). The administration of sodium butyrate, a byproduct of butyrate, induces demethylation, facilitating the restoration of normal epigenetic expression, and highlights the critical role of butyrate fluctuations in TNs severity. An increased presence of the genus *Ruminococcaceae*_NK4A214_group enhances butyrate production, potentially slowing the progression to advanced TNs stages. This study identified a negative causal relationship between *Ruminococcaceae*_NK4A214_group and TNs, suggesting the *Ruminococcaceae* family may mitigate TNs progression through regulatory mechanisms of butyrate production, exerting anti-inflammatory effects, modulating immune responses, and restoring epigenetic expressions. Conversely, this investigation identifies a negative causal relationship between the *Lachnospiraceae* and TNs risk. Bacteria within the *Lachnospiraceae* family are essential for maintaining gut health and the balance of the host immune system by fermenting dietary fibers to produce short-chain fatty acids (SCFAs), including butyrate, affecting the host’s digestion and metabolism (39). SCFAs not only serve as an energy source but also regulate hormone secretion, such as insulin. Alterations in *Lachnospiraceae* have been associated to the development of obesity and type 2 diabetes—both recognized risk factors for TNs (40, 41). Hence, a reduction in *Lachnospiraceae* bacteria leads to GM imbalance, notably a reduction in butyrate-producing bacteria, impacting the metabolic and immune regulation of the thyroid-gut axis, corroborating the findings of Ang Li et al. (21).

*Senegalimassilia*, characterized as a class of anaerobic probiotics involved in cellulose breakdown, has a significant impact on maintaining gut health and immune system balance. The prevalence of *Senegalimassilia* in the gut has been associated with the progression of various conditions, including non-alcoholic fatty liver disease, bladder cancer, pancreatic cancer, and polycystic ovary syndrome (42–45). Research indicates that metformin treatment enhances the abundance of *Senegalimassilia* in the guts of young, healthy males, correlating with decreased thyroid volumes and reduced risks of thyroid enlargement, TNs, and cancer in treated patients (46). The increase in *Senegalimassilia* may confer a protective effect on the thyroid via the thyroid-intestine axis, warranting further investigation into this intricate relationship.

*Desulfovibrionales*, classified within the δ-Proteobacteria class, are implicated in an array of conditions, including psychiatric and gastrointestinal disorders. Research has demonstrated that *Desulfovibrionales* foster cholesterol gallstone formation by augmenting secondary bile acid production in the cecum, which increases the hydrophobicity of bile acids and regulating the absorption and secretion of intestinal cholesterol (47). The interconnection between thyroid disorders, including thyroid nodules (TNs), and cholelithiasis is established; thyroid pathologies are significant risk factors for gallstones (48), with the influence of thyroid hormones on nuclear factors and cholesterol transport proteins playing a critical role in cholesterol homeostasis and the progression of cholelithiasis (49). Intestinal epithelial cells, capable of responding to signals from pathogenic and commensal bacteria such as secondary bile acids, relay these signals to gut-resident innate immune cells (50). This process promotes the translocation of bacteria and their metabolites through the intestinal barrier into the bloodstream, triggering persistent inflammatory stimulation and initiating immune responses. Chronic inflammation plays a crucial role in TNs, with elevated leukocyte counts serving as independent predictors for TNs. It is proposed that the type 2 immune response, marked by an increase in eosinophil percentages, is associated with TNs presence (51). Our results indicate a positive causal relationship between *Desulfovibrionales* and TNs, suggesting that enrichment of *Desulfovibrionales* may provoke inflammatory responses by altering nutrient metabolism, thus intensifying immune reactions and increasing TNs risk.

*Prevotella*, an anaerobic Gram-negative genus within the order Bacteroidales and family *Prevotellaceae*, encompasses *Prevotella*_7, a dominant intestinal bacterium widespread in the human gut. Evidence indicates that a low proportion of *Prevotella* may beneficially affect gut microbial biodiversity balance (52). *Prevotella* enrichment has been observed to disrupt the Th1/Th2 cytokine equilibrium, elevating cytokine levels such as TNF-α and IFN-γ, potentially causing thyroid damage (53). Additionally, there is considerable evidence of parallels between oral microbiota and GM (54, 55). Investigations into oral microbiota have shown that unclassified *Prevotellaceae* may accelerate TNs progression, with increased *Alloprevotella* abundance in saliva linked to thyroid cancer; *Alloprevotella* belongs to the *Prevotella* genus (56). Analyses of GM in TC patients also identified significant dysbiosis in *Prevotellaceae* and *Prevotella*_9 among others (57). Our study posits a positive causal association between *Prevotella*_7 and TNs, potentially due to *Prevotella*_7 enrichment compromising intestinal barrier integrity, exacerbating intestinal inflammation, and affecting signaling pathways involved in systemic immune responses, thereby influencing thyroid health (58–60).

*Deltaproteobacteria* are a group of Gram-negative Proteobacteria consisting of obligate anaerobes, including several sulfate-reducing and sulfur-reducing bacteria (61). Although generally present in low abundance in healthy individuals, they may play significant roles in specific pathological conditions. A strong causal relationship between *Deltaproteobacteria* and thyroid function, particularly hyperthyroidism, has been established, with studies identifying *Deltaproteobacteria* as a risk factor for Graves’ disease (62); Cao J et al. highlighted *Deltaproteobacteria* as a potential risk factor (63). *Desulfovibrionaceae*, within the *Deltaproteobacteria* class, decomposes sulfur-containing compounds in the gastrointestinal tract into hydrogen sulfide. At elevated concentrations, hydrogen sulfide can harm intestinal epithelial cells, induce DNA damage, and exert pro-inflammatory effects, correlating with various diseases (64). Overgrowth of *Desulfovibrionaceae* has been associated with colitis and colorectal cancer (65). Furthermore, *Desulfovibrionaceae* impact hormone metabolism, including sex and thyroid hormones. Shen H et al. observed that iodine supplementation resulted in decreased serum thyroid hormone levels in female mice, with significant GM composition shifts, notably an increase in *Desulfovibrionaceae*, suggesting a higher thyroid disease risk in females associated with *Desulfovibrionaceae* proliferation (66). The absorption of essential thyroid minerals, such as iodine and selenium, is closely linked to *Deltaproteobacteria* and *Desulfovibrionaceae*, influencing TNs development. Our research indicates a positive causal link between *Deltaproteobacteria*, *Desulfovibrionaceae*, and TNs, suggesting their enrichment may elevate TNs risk, potentially alongside thyroid function disturbances.

*Faecalibacterium*, recognized for its probiotic properties, plays a important ecological role in the human gut. Its functions include the synthesis of anti-inflammatory metabolites such as butyrate and salicylate, safeguarding the digestive tract from pathogenic threats (67). In patients with Hashimoto’s thyroiditis, a decline in *Faecalibacterium* prausnitzii abundance has been noted with an inverse correlation between *Faecalibacterium*’s relative abundance and thyroid autoantibodies or the FT3 to FT4 ratio, underscoring a close association with thyroid hormones and their functionality (21, 68, 69). Nonetheless, our findings suggest a positive correlation between *Faecalibacterium* and TNs incidence, raising queries about whether *Faecalibacterium* overproliferation leading to gut microbiota dysbiosis is implicated, necessitating further investigation.

In essence, the prevalence of various GM in patients with TNs alters as the disease progresses, and reciprocally, the GM can influence the onset and development of TNs through mechanisms such as regulation of glycolipid metabolism, mitigation of intestinal inflammation, participation in immune responses, modulation of the intestinal barrier, and provision of gut nutrition.

However, this study is not devoid of limitations. Primarily, the GWAS statistical data were derived solely from European populations, potentially limiting the generalizability of our findings. Additionally, the application of stringent p-value thresholds (*p*< 1×10^-5^) might have introduced potential false-positive associations. Moreover, the lack of individual-level data from the GWAS prevented detailed patient stratification analysis, restricting the exploration of interactions between the GM and various TNs patient stratifications. Despite these limitations, this study, through comprehensive MR analyses, provides supportive evidence for a causal relationship between the GM and TNs, emphasizing the importance of the thyroid-gut axis. These insights pave the way for future studies in broader populations, potentially heralding novel diagnostic and therapeutic interventions.

## 5 Conclusion

In this study, the causal relationship between the GM and TNs were evaluated using univariate and multivariate MR analyses. Our findings suggest that certain microbiota, identified as *Ruminococcaceae*_NK4A214_group, *Senegalimassilia*, *Lachnospiraceae*, exhibit a protective influence against TNs’ development, indicated by negative causal associations. In contrast, microbiota categorized as *Desulfovibrionales*, *Prevotella*_7, *Faecalibacterium*, *Desulfovibrionaceae*, *Deltaproteobacteria* are positively associated with TNs, suggesting they may serve as risk factors. Reverse MR analyses did not establish significant causal links. After comprehensive adjustment for confounders, taxa *Desulfovibrionales* (Order), *Desulfovibrionaceae* (Family), *Deltaproteobacteria* (Class) remain implicated as potential contributors to TNs’ risk. Future studies are warranted to further substantiate the GM-TNs causal relationship, elucidate underlying mechanisms, and enrich preventive and therapeutic strategies in clinical settings.

## Data availability statement

The original contributions presented in the study are included in the article/**Supplementary Material**. Further inquiries can be directed to the corresponding authors.

## Author contributions

SY: Writing – original draft, Writing – review & editing. JH: Writing – original draft, Writing – review & editing. XY: Writing – original draft, Writing – review & editing. JS: Writing – original draft, Writing – review & editing. YZ: Writing – original draft, Writing – review & editing. HB: Writing – original draft, Writing – review & editing. XZ: Writing – original draft, Writing – review & editing. XX: Writing – original draft, Writing – review & editing. LL: Writing – original draft, Writing – review & editing.

## References

[1] Sung H Ferlay J Siegel RL Laversanne M Soerjomataram I Jemal A. Global cancer statistics 2020: GLOBOCAN estimates of incidence and mortality worldwide for 36 cancers in 185 countries. CA Cancer J Clin. (2021) 71:209–49. doi: 10.3322/caac.21660 33538338

[2] Jiang H Tian Y Yan W Kong Y Wang H Wang A. The prevalence of thyroid nodules and an analysis of related lifestyle factors in Beijing communities. Int J Environ Res Public Health. (2016) 13:442. doi: 10.3390/ijerph13040442 27110805 PMC4847104

[3] Popoveniuc G Jonklaas J. Thyroid nodules. Med Clin North Am. (2012) 96:329–49. doi: 10.1016/j.mcna.2012.02.002 PMC3575959 22443979

[4] Uppal N Collins R James B. Thyroid nodules: Global, economic, and personal burdens. Front Endocrinol (Lausanne). (2023) 14:1113977. doi: 10.3389/fendo.2023.1113977 36755911 PMC9899850

[5] Yan Y Dong J Li S Yang G Huang K Tian W. Risk factors associated with the prevalence of thyroid nodules in adults in Northeast China: a cross-sectional population-based study. BMJ Open. (2023) 13:e069390. doi: 10.1136/bmjopen-2022-069390 PMC10619099 37907298

[6] Ahmadi S Pappa T Kang AS Coleman AK Landa I Marqusee E. Point of care measurement of body mass index and thyroid nodule Malignancy risk assessment. Front Endocrinol (Lausanne). (2022) 13:824226. doi: 10.3389/fendo.2022.824226 35222281 PMC8873520

[7] Lozupone CA Stombaugh JI Gordon JI Jansson JK Knight R. Diversity, stability and resilience of the human gut microbiota. Nature. (2012) 489:220–30. doi: 10.1038/nature11550 PMC3577372 22972295

[8] Adak A Khan MR. An insight into gut microbiota and its functionalities. Cell Mol Life Sci. (2019) 76:473–93. doi: 10.1007/s00018-018-2943-4 PMC11105460 30317530

[9] Fung TC Olson CA Hsiao EY. Interactions between the microbiota, immune and nervous systems in health and disease. Nat Neurosci. (2017) 20:145–55. doi: 10.1038/nn.4476 PMC6960010 28092661

[10] Tang WH Kitai T Hazen SL. Gut microbiota in cardiovascular health and disease. Circ Res. (2017) 120:1183–96. doi: 10.1161/CIRCRESAHA.117.309715 PMC5390330 28360349

[11] Budden KF Gellatly SL Wood DL Cooper MA Morrison M Hugenholtz P. Emerging pathogenic links between microbiota and the gut-lung axis. Nat Rev Microbiol. (2017) 15:55–63. doi: 10.1038/nrmicro.2016.142 27694885

[12] Knezevic J Starchl C Tmava Berisha A Amrein K. Thyroid-gut-axis: how does the microbiota influence thyroid function? Nutrients. (2020) 12:1769. doi: 10.3390/nu12061769 32545596 PMC7353203

[13] Ishaq HM Mohammad IS Guo H Shahzad M Hou YJ Ma C. Molecular estimation of alteration in intestinal microbial composition in Hashimoto's thyroiditis patients. BioMed Pharmacother. (2017) 95:865–74. doi: 10.1016/j.biopha.2017.08.101 28903182

[14] Virili C Fallahi P Antonelli A Benvenga S Centanni M. Gut microbiota and Hashimoto's thyroiditis. Rev Endocr Metab Disord. (2018) 19:293–300. doi: 10.1007/s11154-018-9467-y 30294759

[15] Yu X Jiang W Kosik RO Song Y Luo Q Qiao T. Gut microbiota changes and its potential relations with thyroid carcinoma. J Adv Res. (2021) 35:61–70. doi: 10.1016/j.jare.2021.04.001 35003794 PMC8721249

[16] Hou J Tang Y Chen Y Chen D. The role of the microbiota in graves' Disease and graves' Orbitopathy. Front Cell Infect Microbiol. (2021) 11:739707. doi: 10.3389/fcimb.2021.739707 35004341 PMC8727912

[17] Su X Zhao Y Li Y Ma S Wang Z. Gut dysbiosis is associated with primary hypothyroidism with interaction on gut-thyroid axis. Clin Sci (Lond). (2020) 134:1521–35. doi: 10.1042/CS20200475 32519746

[18] Fröhlich E Wahl R. Microbiota and thyroid interaction in health and disease. Trends Endocrinol Metab. (2019) 30:479–90. doi: 10.1016/j.tem.2019.05.008 31257166

[19] Shen H Han J Li Y Lu C Zhou J Li Y. Different host-specific responses in thyroid function and gut microbiota modulation between diet-induced obese and normal mice given the same dose of iodine. Appl Microbiol Biotechnol. (2019) 103:3537–47. doi: 10.1007/s00253-019-09687-1 30850874

[20] Shi N Li N Duan X Niu H. Interaction between the gut microbiome and mucosal immune system. Mil Med Res. (2017) 4:14. doi: 10.1186/s40779-017-0122-9 28465831 PMC5408367

[21] Li A Li T Gao X Yan H Chen J Huang M. Gut microbiome alterations inpatients with thyroid nodules. Front Cell Infect Microbiol. (2021) 11:643968. doi: 10.3389/fcimb.2021.643968 PMC8005713 33791245

[22] Greenberg VL Williams JM Boghaert E Mendenhall M Ain KB Zimmer SG. Butyrate alters the expression and activity of cell cycle components in anaplastic thyroid carcinoma cells. Thyroid. (2001) 11:21–9. doi: 10.1089/10507250150500621 11272092

[23] Sanderson E Glymour MM Holmes MV Kang H Morrison J Munafò MR. Mendelian randomization. Nat Rev Methods Primers. (2022) 2:6. doi: 10.1038/s43586-021-00092-5 37325194 PMC7614635

[24] Emdin CA Khera AV Kathiresan S. Mendelian randomization. JAMA. (2017) 318:1925–6. doi: 10.1001/jama.2017.17219 29164242

[25] Davies NM Holmes MV Davey Smith G. Reading Mendelian randomisation studies: a guide, glossary, and checklist for clinicians. BMJ. (2018) 362:k601. doi: 10.1136/bmj.k601 30002074 PMC6041728

[26] Skrivankova VW Richmond RC Woolf BAR Yarmolinsky J Davies NM Swanson SA. Strengthening the reporting of observational studies in epidemiology using mendelian randomization: The STROBE-MR statement. JAMA. (2021) 326:1614–21. doi: 10.1001/jama.2021.18236 34698778

[27] Kurilshikov A Medina-Gomez C Bacigalupe R Radjabzadeh D Wang J Demirkan A. Large-scale association analyses identify host factors influencing human gut microbiome composition. Nat Genet. (2021) 53:156–65. doi: 10.1038/s41588-020-00763-1 PMC8515199 33462485

[28] Liu X Qi X Han R Mao T Tian Z. Gut microbiota causally affects cholelithiasis: a two-sample Mendelian randomization study. Front Cell Infect Microbiol. (2023) 13:1253447. doi: 10.3389/fcimb.2023.1253447 37876873 PMC10591199

[29] Sanna S van Zuydam NR Mahajan A Kurilshikov A Vich Vila A Võsa U. Causal relationships among the gut microbiome, short-chain fatty acids and metabolic diseases. Nat Genet. (2019) 51:600–5. doi: 10.1038/s41588-019-0350-x PMC6441384 30778224

[30] Li P Wang H Guo L Gou X Chen G Lin D. Association between gut microbiota and preeclampsia-eclampsia: a two-sample Mendelian randomization study. BMC Med. (2022) 20:443. doi: 10.1186/s12916-022-02657-x 36380372 PMC9667679

[31] Choi KW Chen CY Stein MB Klimentidis YC Wang MJ Koenen KC. Assessment of Bidirectional Relationships Between Physical Activity and Depression Among Adults: A 2-Sample Mendelian Randomization Study (published correction appears in JAMA Psychiatry. 2023 Oct 1;80(10):1078). JAMA Psychiatry. (2019) 76:399–408. doi: 10.1001/jamapsychiatry.2018.4175 30673066 PMC6450288

[32] Yin KJ Huang JX Wang P Yang XK Tao SS Li HM. No genetic causal association between periodontitis and arthritis: A bidirectional two-sample mendelian randomization analysis. Front Immunol. (2022) 13:808832. doi: 10.3389/fimmu.2022.808832 35154127 PMC8825874

[33] Boehm FJ Zhou X. Statistical methods for Mendelian randomization in genome-wide association studies: A review. Comput Struct Biotechnol J. (2022) 20:2338–51. doi: 10.1016/j.csbj.2022.05.015 PMC9123217 35615025

[34] Chen M Peng WY Tang TC Zheng H. Differential sleep traits have no causal effect on inflammatory bowel diseases: A mendelian randomization study. Front Pharmacol. (2021) 12:763649. doi: 10.3389/fphar.2021.763649 34916940 PMC8669049

[35] Gao Y Fan ZR Shi FY. Hypothyroidism and rheumatoid arthritis: a two-sample Mendelian randomization study. Front Endocrinol (Lausanne). (2023) 14:1179656. doi: 10.3389/fendo.2023.1179656 37324262 PMC10262846

[36] Di J Ge Z Xie Q Kong D Liu S Wang P. Helicobacter pylori infection increases the risk of thyroid nodules in adults of Northwest China. Front Cell Infect Microbiol. (2023) 13:1134520. doi: 10.3389/fcimb.2023.1134520 37065186 PMC10102366

[37] Wang L Liao Y Yang R Zhu Z Zhang L Wu Z. An engineered probiotic secreting Sj16 ameliorates colitis via Ruminococcaceae/butyrate/retinoic acid axis. Bioeng Transl Med. (2021) 6:e10219. doi: 10.1002/btm2.10219 34589596 PMC8459592

[38] Yim JH Choi AH Li AX Qin H Chang S Tong ST. Identification of tissue-specific DNA methylation signatures for thyroid nodule diagnostics. Clin Cancer Res. (2019) 25:544–51. doi: 10.1158/1078-0432.CCR-18-0841 PMC6335179 30093451

[39] Yang YH Chen C Zheng Y Wu ZJ Zhou MQ Liu XY. Fucoxanthin alleviates dextran sulfate sodium-induced colitis and gut microbiota dysbiosis in mice. J Agric Food Chem. (2024) 72:4142–54. doi: 10.1021/acs.jafc.3c08811 38355398

[40] Zeng L Ma J Wei T Wang H Yang G Han C. The effect of canagliflozin on gut microbiota and metabolites in type 2 diabetic mice. Genes Genomics. (2024) 46(5):541–55. doi: 10.1007/s13258-024-01491-0 38483772

[41] Yamamoto M Ogura H Kuda T Xia Y Nakamura A Takahashi H. Detection of typical indigenous gut bacteria related to kanpyo Lagenaria siceraria var. hispida powder in murine caecum and human faecal cultures. 3 Biotech. (2024) 14:118. doi: 10.1007/s13205-024-03960-5 PMC10959864 38524237

[42] Dai W Cai D Zhou S Li A Xie J Zhang J. Uncovering a causal connection between the Lachnoclostridium genus in fecal microbiota and non-alcoholic fatty liver disease: a two-sample Mendelian randomization analysis. Front Microbiol. (2023) 14:1276790. doi: 10.3389/fmicb.2023.1276790 38192292 PMC10773585

[43] Yin Z Liu B Feng S He Y Tang C Chen P. A large genetic causal analysis of the gut microbiota and urological cancers: A bidirectional mendelian randomization study. Nutrients. (2023) 15:4086. doi: 10.3390/nu15184086 37764869 PMC10537765

[44] Jiang Z Mou Y Wang H Li L Jin T Wang H. Causal effect between gut microbiota and pancreatic cancer: a two-sample Mendelian randomization study. BMC Cancer. (2023) 23:1091. doi: 10.1186/s12885-023-11493-y 37950180 PMC10636952

[45] Garcia-Beltran C Malpique R Carbonetto B González-Torres P Henares D Brotons P. Gut microbiota in adolescent girls with polycystic ovary syndrome: Effects of randomized treatments. Pediatr Obes. (2021) 16:e12734. doi: 10.1111/ijpo.12734 32989872

[46] Meng X Xu S Chen G Derwahl M Liu C. Metformin and thyroid disease. J Endocrinol. (2017) 233:R43–51. doi: 10.1530/JOE-16-0450 28196954

[47] Hu H Shao W Liu Q Liu N Wang Q Xu J. Gut microbiota promotes cholesterol gallstone formation by modulating bile acid composition and biliary cholesterol secretion. Nat Commun. (2022) 13:252. doi: 10.1038/s41467-021-27758-8 35017486 PMC8752841

[48] Song ST Shi J Wang XH Guo YB Hu PF Zhu F. Prevalence and risk factors for gallstone disease: A population-based cross-sectional study. J Dig Dis. (2020) 21:237–45. doi: 10.1111/1751-2980.12857 32166900

[49] Ravi PC Thugu TR Singh J Dasireddy RR Kumar SA Isaac NV. Gallstone disease and its correlation with thyroid disorders: A narrative review. Cureus. (2023) 15:e45116. doi: 10.7759/cureus.45116 37842424 PMC10568238

[50] Kayama H Okumura R Takeda K. Interaction between the microbiota, epithelia, and immune cells in the intestine. Annu Rev Immunol. (2020) 26:23–48. doi: 10.1146/annurev-immunol-070119-115104 32340570

[51] Wang S Wang X Hua X Jiang S Xie Y Liu H. Adjusted association between type 2 immunity and low risk thyroid nodules: a retrospective cohort study. BMC Endocr Disord. (2022) 22:2. doi: 10.1186/s12902-021-00917-0 34983483 PMC8725489

[52] Liang Y Yu W Wang H Yao L He Z Sun M. Flash extraction of ulvan polysaccharides from marine green macroalga Ulva linza and evaluation of its antioxidant and gut microbiota modulation activities. Int J Biol Macromol. (2024) 262:130174. doi: 10.1016/j.ijbiomac.2024.130174 38360235

[53] Wu B Xu Y Ban Y Zhang M Sun Z Cai Y. Correlation between the intestinal microflora and peripheral blood Th1/Th2 balance in hypothyroidism during the first half of pregnancy. Front Cell Infect Microbiol. (2023) 13:1159238. doi: 10.3389/fcimb.2023.1159238 37051293 PMC10083372

[54] Maki KA Kazmi N Barb JJ Ames N. The oral and gut bacterial microbiomes: Similarities, differences, and connections. Biol Res Nurs. (2021) 23:7–20. doi: 10.1177/1099800420941606 32691605 PMC8822203

[55] Campisciano G Zanotta N Quadrifoglio M Careri A Torresani A Cason C. The bacterial DNA profiling of chorionic villi and amniotic fluids reveals overlaps with maternal oral, vaginal, and gut microbiomes. Int J Mol Sci. (2023) 24:2873. doi: 10.3390/ijms24032873 36769194 PMC9917689

[56] Jiao J Zheng Y Zhang Q Xia D Zhang L Ma N. Saliva microbiome changes in thyroid cancer and thyroid nodules patients. Front Cell Infect Microbiol. (2022) 12:989188. doi: 10.3389/fcimb.2022.989188 36034695 PMC9403763

[57] Ishaq HM Mohammad IS Hussain R Parveen R Shirazi JH Fan Y. Gut-Thyroid axis: How gut microbial dysbiosis associated with euthyroid thyroid cancer. J Cancer. (2022) 13:2014–28. doi: 10.7150/jca.66816 PMC8990431 35399732

[58] Sun L Jia H Li J Yu M Yang Y Tian D. Cecal gut microbiota and metabolites might contribute to the severity of acute myocardial ischemia by impacting the intestinal permeability, oxidative stress, and energy metabolism. Front Microbiol. (2019) 10:1745. doi: 10.3389/fmicb.2019.01745 31428065 PMC6687875

[59] Feng W Yang Z Liu Y Chen R Song Z Pan G. Gut microbiota: A new target of traditional Chinese medicine for insomnia. BioMed Pharmacother. (2023) 160:114344. doi: 10.1016/j.biopha.2023.114344 36738504

[60] Chen Y Liu Y Wang Y Chen X Wang C Chen X. Prevotellaceae produces butyrate to alleviate PD-1/PD-L1 inhibitor-related cardiotoxicity via PPARα-CYP4X1 axis in colonic macrophages. J Exp Clin Cancer Res. (2022) 41:1. doi: 10.1186/s13046-021-02201-4 34980222 PMC8722009

[61] Totzeck AA-O Ramakrishnan E Schlag M Stolte B Kizina K Bolz S. Gut bacterial microbiota in patients with myasthenia gravis: results from the MYBIOM study. Ther Adv Neurol Disord. (2021) 14:17562864211035657. doi: 10.1177/17562864211035657 34394728 PMC8361534

[62] Xie L Zhao H Chen W. Relationship between gut microbiota and thyroid function: a two-sample Mendelian randomization study. Front Endocrinol (Lausanne). (2023) 14:1240752. doi: 10.3389/fendo.2023.1240752 37822602 PMC10562735

[63] Cao J Wang N Luo Y Ma C Chen Z Chenzhao C. A cause-effect relationship between Graves' disease and the gut microbiome contributes to the thyroid-gut axis: A bidirectional two-sample Mendelian randomization study. Front Immunol. (2023) 14:977587. doi: 10.3389/fimmu.2023.977587 36865531 PMC9974146

[64] Ma F Sun M Song Y Wang A Jiang S Qian F. Lactiplantibacillus plantarum-12 Alleviates Inflammation and Colon Cancer Symptoms in AOM/DSS-Treated Mice through Modulating the Intestinal Microbiome and Metabolome. Nutrients. (2022) 14:1916. doi: 10.3390/nu14091916 35565884 PMC9100115

[65] Millien V Rosen D Hou J Shah R. Proinflammatory sulfur-reducing bacteria are more abundant in colonic biopsies of patients with microscopic colitis compared to healthy controls. Dig. Dis Sci. (2019) 64:432–8. doi: 10.1007/s10620-018-5313-z 30324555

[66] Shen H Xu J Lu C Han J Zhou J Ming T. Effects of the sex factor on mouse iodine intake: Interactions between the gut microbiota composition and metabolic syndromes. ACS Omega. (2021) 6:28569–78. doi: 10.1021/acsomega.1c02697 PMC8567277 34746552

[67] Martín R Rios-Covian D Huillet E Auger S Khazaal S Bermúdez-Humarán LG. Faecalibacterium: a bacterial genus with promising human health applications. FEMS Microbiol Rev. (2023) 47:fuad039. doi: 10.1093/femsre/fuad039 37451743 PMC10410495

[68] Gong B Wang C Meng F Wang H Song B Yang Y. Association between gut microbiota and autoimmune thyroid disease: A systematic review and meta-analysis. Front Endocrinol (Lausanne). (2021) 12:774362. doi: 10.3389/fendo.2021.774362 34867823 PMC8635774

[69] Liu J Qin X Lin B Cui J Liao J Zhang F. Analysis of gut microbiota diversity in Hashimoto's thyroiditis patients. BMC Microbiol. (2022) 22:318. doi: 10.1186/s12866-022-02739-z 36564707 PMC9789560

