# Supplementary material for: Causal relationship between gut microbiota and thyroid nodules: a bidirectional two-sample Mendelian randomization study
Source: Front Endocrinol (Lausanne). 2024 Aug 8;15:1417009. doi: 10.3389/fendo.2024.1417009 (PMC11338761; doi:10.3389/fendo.2024.1417009)
Supplement: Supplementary file 1 [file Table_1.docx]

Catalogue

[Supplementary Table 1 STROBE-MR Checklist (Skrivankova et al., 2021) 3](#_Toc171353130)

[Supplementary Table 2 Data details for GWAS 12](#_Toc171353131)

[Supplementary Table 3 The SNPs included according to selection criteria as instrumental variables for gut microbiota and thyroid nodules 13](#_Toc171353132)

[Supplementary Table 4 Mendelian randomization analysis results between gut microbiota and thyroid nodules 18](#_Toc171353133)

[Supplementary Table 5 Heterogeneity and Pleiotropy tests for MR analysis of gut microbiota and thyroid nodules 22](#_Toc171353134)

[Supplementary Table 6 MR-PRESSO analysis for the association between gut microbiota and thyroid nodules 23](#_Toc171353135)

[Supplementary Table 7 The Leave-one-out analysis for IVW MR of gut microbiota on thyroid nodules 24](#_Toc171353136)

[Supplementary Table 8 The SNPs included according to selection criteria as instrumental variables for thyroid nodules on gut microbiota 29](#_Toc171353137)

[Supplementary Table 9 Causal estiamte from thyroid nodules exposure to identified microbiota outcomes in the reverse MR analysis 32](#_Toc171353138)

[Supplementary Table 10 Heterogeneity and Pleiotropy tests for reverse MR analysis of thyroid nodules on gut microbiota 34](#_Toc171353139)

[Supplementary Table 11 Leave-one-out analysis for IVW MR of thyroid nodules on gut microbiota 35](#_Toc171353140)

[Supplementary Table 12 Multivariable mendelian randomization analysis results between gut micriobiota and thyroid nodules (adjusting five variables separately) 38](#_Toc171353141)

[Supplementary Table 13 Multivariable mendelian randomization analysis results between gut microbiota and thyroid nodules (adjusting five variables) 48](#_Toc171353142)

[Supplementary Table 14 Heterogeneity and Pleiotropy tests for multivariable MR analysis of gut microbiota and thyroid nodules 51](#_Toc171353143)

# Supplementary Table 1 STROBE-MR Checklist (Skrivankova et al., 2021)

| **Item No.** | **Section** | **Checklist item** | **Manuscript section and paragraph** |
| --- | --- | --- | --- |
| **Title and abstract** |  |  |  |
| 1 | Title and abstract | Indicate mendelian randomization (MR) as the study’s design in the title and/or the abstract if that is a main purpose of the study. | Title and abstract |
| **Introduction** |  |  |  |
| 2 | Background | Explain the scientific background and rationale for the reported study. What is the exposure? Is a potential causal relationship between exposure and outcome plausible? Justify why MR is a helpful method to address the study question. | Introduction, paragraphs 1-3 |
| 3 | Objectives | State specific objectives clearly, including prespecified causal hypotheses (if any). State that MR is a method that, under specific assumptions, intends to estimate causal effects. | Introduction, paragraphs 4 |
| **Methods** |  |  |  |
| 4 | Study design and data sources | Present key elements of the study design early in the article. Consider including a table listing sources of data for all phases of the study. For each data source contributing to the analysis, describe the following: | Methods; Figure 1 |
|  | a | Setting: Describe the study design and the underlying population, if possible. Describe the setting, locations, and relevant dates, including periods of recruitment, exposure, follow-up, and data collection, when available. | Methods, "Ethics Statement and study design" section |
|  | b | Participants: Report the eligibility criteria and the sources and methods of selection of participants. Report the sample size and whether any power or sample size calculations were carried out prior to the main analysis. | Methods, "Data source" section and Supplementary Table 2 |
|  | c | Describe measurement, quality control, and selection of genetic variants. | Methods, "Selection of Instrumental variable" section |
|  | d | For each exposure, outcome, and other relevant variables, describe methods of assessment and diagnostic criteria for diseases. | Methods, "Data source and Selection of Instrumental variable" section |
|  | e | Provide details of ethics committee approval and participant informed consent, if relevant. | Methods, "Ethics Statement and study design" section |
| 5 | Assumptions | Explicitly state the 3 core instrumental variable (IV) assumptions for the main analysis (relevance, independence, and exclusion restriction), as well assumptions for any additional or sensitivity analysis. | Methods, "Ethics Statement and study design" section and Figure 1 |
| 6 | Statistical methods: main analysis | Describe statistical methods and statistics used. | Methods, "Statistical Analysis of Mendelian Randomization" sections |
|  | a | Describe how quantitative variables were handled in the analyses (ie, scale, units, model). | Methods, "Statistical Analysis of Mendelian Randomization" sections |
|  | b | Describe how genetic variants were handled in the analyses and, if applicable, how their weights were selected. | Methods, "Statistical Analysis of Mendelian Randomization" sections |
|  | c | Describe the MR estimator (eg, 2-stage least squares, Wald ratio) and related statistics. Detail the included covariates and, in case of 2-sample MR, whether the same covariate set was used for adjustment in the 2 samples. | Methods, "Statistical Analysis of Mendelian Randomization" sections |
|  | d | Explain how missing data were addressed. | N/A |
|  | e | If applicable, indicate how multiple testing was addressed. | Methods, "Statistical Analysis of Mendelian Randomization" sections |
| 7 | Assessment of assumptions | Describe any methods or prior knowledge used to assess the assumptions or justify their validity. | N/A |
| 8 | Sensitivity analyses and additional analyses | Describe any sensitivity analyses or additional analyses performed (eg, comparison of effect estimates from different approaches, independent replication, bias analytic techniques, validation of instruments, simulations). | Methods, "Statistical Analysis of Mendelian Randomization" sections |
| 9 | Software and preregistration |  |  |
|  | a | Name statistical software and package(s), including version and settings used. | Methods, "Statistical Analysis of Mendelian Randomization" sections |
|  | b | State whether the study protocol and details were preregistered (as well as when and where). | N/A |
| **Results** |  |  |  |
| 10 | Descriptive data |  |  |
|  | a | Report the numbers of individuals at each stage of included studies and reasons for exclusion. Consider use of a flow diagram. | Results, "Selection of instrumental variables,"Figure 2" |
|  | b | Report summary statistics for phenotypic exposure(s), outcome(s), and other relevant variables (eg, means, SDs, proportions). | Supplementary Table 3, Supplementary Table 8 |
|  | c | If the data sources include meta-analyses of previous studies, provide the assessments of heterogeneity across these studies. | N/A |
|  | d | For 2-sample MR: i. Provide justification of the similarity of the genetic variant–exposure associations between the exposure and outcome samples. ii. Provide information on the number of individuals who overlap between the exposure and outcome studies. | N/A |
| 11 | Main results |  |  |
|  | a | Report the associations between genetic variant and exposure and between genetic variant and outcome, preferably on an interpretable scale. | Results, "Causal effects of gut microbiota on thyroid nodules by UVMR", "Causal effects of thyroid nodules on gut microbiota" and "Causal effects of exposure on thyroid nodules by MVMR" sections |
|  | b | Report MR estimates of the relationship between exposure and outcome and the measures of uncertainty from the MR analysis, on an interpretable scale, such as odds ratio or relative risk per SD difference. | Supplementary Table 4,and Supplementary Table 9,Supplementary Table 12 |
|  | c | If relevant, consider translating estimates of relative risk into absolute risk for a meaningful time period. | N/A |
|  | d | Consider plots to visualize results (eg, forest plot, scatterplot of associations between genetic variants and outcome vs between genetic variants and exposure). | Figure 2, 3, 4, 5 |
| 12 | Assessment of assumptions |  |  |
|  | a | Report the assessment of the validity of the assumptions. | Results, Supplementary Table 3, Supplementary Table 8 |
|  | b | Report any additional statistics (eg, assessments of heterogeneity across genetic variants, such as I2, Q statistic, or E-value). | Results, Supplementary Table 3, Supplementary Table 8 |
| 13 | Sensitivity analyses and additional analyses |  |  |
|  | a | Report any sensitivity analyses to assess the robustness of the main results to violations of the assumptions. | Results, Supplementary Table 5, 6,and 7; Supplementary Table 10,11 and 14 |
|  | b | Report results from other sensitivity analyses or additional analyses. | Results, Supplementary Table 5, 6,and 7; Supplementary Table 10,11 and 14 |
|  | c | Report any assessment of the direction of the causal relationship (eg, bidirectional MR). | Supplementary Table 9,10 |
|  | d | When relevant, report and compare with estimates from non-MR analyses. | N/A |
|  | e | Consider additional plots to visualize results (eg, leave-one-out analyses). | N/A |
| **Discussion** |  |  |  |
| 14 | Key results | Summarize key results with reference to study objectives. | Discussion, paragraph 2 |
| 15 | Limitations | Discuss limitations of the study, taking into account the validity of the IV assumptions, other sources of potential bias, and imprecision. Discuss both direction and magnitude of any potential bias and any efforts to address them. | Discussion, paragraph 10 |
| 16 | Interpretation |  |  |
|  | a | Meaning: Give a cautious overall interpretation of results in the context of their limitations and in comparison with other studies. | Discussion, paragraph 3-9 |
|  | b | Mechanism: Discuss underlying biological mechanisms that could drive a potential causal relationship between the investigated exposure and the outcome, and whether the gene-environment equivalence assumption is reasonable. Use causal language carefully, clarifying that IV estimates may provide causal effects only under certain assumptions. | Discussion, paragraph 3-9 |
|  | c | Clinical relevance: Discuss whether the results have clinical or public policy relevance, and to what extent they inform effect sizes of possible interventions. | Discussion, paragraph 3-9 |
| 17 | Generalizability | Discuss the generalizability of the study results (a) to other populations, (b) across other exposure periods/timings, and (c) across other levels of exposure. | Discussion, paragraph 10 |
| **Other Information** |  |  |  |
| 18 | Funding | Describe sources of funding and the role of funders in the present study and, if applicable, sources of funding for the databases and original study or studies on which the present study is based. | Funding |
| 19 | Data and data sharing | Provide the data used to perform all analyses or report where and how the data can be accessed, and reference these sources in the article. Provide the statistical code needed to reproduce the results in the article or report whether the code is publicly accessible and, if so, where. | Data Availability Statement |
| 20 | Conflicts of interest | All authors should declare all potential conflicts of interest. | Conflict of Interest |

|  | **All** | **Female** | **Male** |
| --- | --- | --- | --- |
| Ethnicity | European | | |
| Control / n | 187,684 |  |  |
| Number of individuals / n | 1121 | 902 | 219 |
| Unadjusted prevalence (%) | 0.52 | 0.73 | 0.23 |
| Mean age at first event (years) | 50.64 | 49.06 | 57.15 |

# Supplementary Table 2 Data details for GWAS

**Table A Data details for thyroid nodule**

Note: disease dataset: finn-b-E4_GOITRENOD

**Data details for confounders**

| **Projects** | **Samplize / n** | **Ethnicity** |
| --- | --- | --- |
| High blood pressure | 407,746 | European |
| Obesity class 3 | 50,364 | European |
| Alcohol consumption | 29,540 | European |
| Type 2 diabetes | 655,666 | European |
| Ever smoked | 99,996 | European |

# Supplementary Table 3 The SNPs included according to selection criteria as instrumental variables for gut microbiota and thyroid nodules

| **Disease** | **Taxa** | **Gut microbiota** | **SNP** | **Effect_allele** | **Other_allele** | **Gut micriobiota/Exposure** | | | **Thyroid nodules/Outcome** | | | **F Statistics** |
| --- | --- | --- | --- | --- | --- | --- | --- | --- | --- | --- | --- | --- |
|  |  |  |  |  |  | **Beta** | **SE** | ***P*-value** | **Beta** | **SE** | ***P*-value** |  |
| Thyroid nodules | class | Deltaproteobacteria | rs1035691 | G | A | -0.055 | 0.012 | 0.00000965 | -0.011 | 0.044 | 0.795 | 24.752 |
|  |  |  | rs112381107 | T | C | 0.207 | 0.046 | 0.00000463 | 0.184 | 0.091 | 0.042 | 21.663 |
|  |  |  | rs11599763 | C | T | -0.054 | 0.012 | 0.00000394 | -0.007 | 0.044 | 0.883 | 22.758 |
|  |  |  | rs16851319 | C | G | -0.071 | 0.015 | 0.00000568 | -0.028 | 0.057 | 0.629 | 20.477 |
|  |  |  | rs17084793 | A | G | -0.071 | 0.016 | 0.00000569 | 0.040 | 0.061 | 0.513 | 20.920 |
|  |  |  | rs17791387 | G | A | -0.074 | 0.015 | 0.0000016 | -0.029 | 0.072 | 0.689 | 21.487 |
|  |  |  | rs2692012 | G | A | 0.110 | 0.025 | 0.00000314 | 0.012 | 0.095 | 0.897 | 20.435 |
|  |  |  | rs2838334 | A | G | 0.056 | 0.012 | 0.00000545 | 0.011 | 0.045 | 0.816 | 19.847 |
|  |  |  | rs3935584 | T | C | -0.052 | 0.012 | 0.0000075 | -0.060 | 0.043 | 0.160 | 18.966 |
|  |  |  | rs4506934 | T | C | -0.094 | 0.020 | 0.00000359 | -0.093 | 0.067 | 0.164 | 20.586 |
|  |  |  | rs55744759 | G | A | -0.078 | 0.017 | 0.00000731 | 0.035 | 0.067 | 0.600 | 20.477 |
|  |  |  | rs6058181 | T | C | 0.083 | 0.017 | 0.00000034 | 0.047 | 0.058 | 0.418 | 21.871 |
|  |  |  | rs62020470 | G | A | -0.059 | 0.013 | 0.00000485 | 0.051 | 0.056 | 0.361 | 20.482 |
|  |  |  | rs9928243 | A | C | -0.054 | 0.012 | 0.00000502 | -0.056 | 0.043 | 0.195 | 20.851 |
|  | Order | Desulfovibrionales | rs112381107 | T | C | 0.210 | 0.046 | 0.00000322 | 0.184 | 0.091 | 0.042 | 33.449 |
|  |  |  | rs11599763 | C | T | -0.055 | 0.012 | 0.00000261 | -0.007 | 0.044 | 0.883 | 23.695 |
|  |  |  | rs16851319 | C | G | -0.073 | 0.015 | 0.0000027 | -0.028 | 0.057 | 0.629 | 23.193 |
|  |  |  | rs17791387 | G | A | -0.073 | 0.015 | 0.00000225 | -0.029 | 0.072 | 0.689 | 23.890 |
|  |  |  | rs186073 | C | T | 0.053 | 0.012 | 0.00000874 | -0.015 | 0.044 | 0.725 | 20.024 |
|  |  |  | rs2692012 | G | A | 0.112 | 0.025 | 0.00000227 | 0.012 | 0.095 | 0.897 | 21.303 |
|  |  |  | rs2838334 | A | G | 0.057 | 0.012 | 0.00000417 | 0.011 | 0.045 | 0.816 | 20.538 |
|  |  |  | rs3935584 | T | C | -0.052 | 0.012 | 0.0000072 | -0.060 | 0.043 | 0.160 | 20.646 |
|  |  |  | rs4506934 | T | C | -0.095 | 0.020 | 0.00000243 | -0.093 | 0.067 | 0.164 | 20.321 |
|  |  |  | rs6058181 | T | C | 0.084 | 0.017 | 0.000000253 | 0.047 | 0.058 | 0.418 | 19.826 |
|  |  |  | rs62020470 | G | A | -0.057 | 0.013 | 0.00000751 | 0.051 | 0.056 | 0.361 | 18.293 |
|  |  |  | rs72647048 | C | T | -0.077 | 0.017 | 0.000009 | 0.035 | 0.067 | 0.602 | 19.437 |
|  |  |  | rs9928243 | A | C | -0.054 | 0.012 | 0.00000397 | -0.056 | 0.043 | 0.195 | 20.791 |
|  | Family | Lachnospiraceae | rs10402491 | C | T | 0.066 | 0.015 | 0.00000758 | -0.027 | 0.058 | 0.637 | 26.279 |
|  |  |  | rs11139361 | T | C | 0.049 | 0.011 | 0.00000426 | -0.082 | 0.046 | 0.073 | 22.022 |
|  |  |  | rs112040820 | A | G | 0.055 | 0.012 | 0.00000242 | 0.029 | 0.049 | 0.561 | 23.534 |
|  |  |  | rs11755180 | C | G | -0.049 | 0.010 | 0.00000269 | -0.043 | 0.043 | 0.320 | 22.727 |
|  |  |  | rs11841382 | G | T | -0.072 | 0.017 | 0.00000958 | 0.046 | 0.077 | 0.550 | 22.300 |
|  |  |  | rs11979110 | T | C | -0.050 | 0.011 | 0.00000182 | -0.017 | 0.043 | 0.685 | 22.030 |
|  |  |  | rs1205443 | A | G | 0.050 | 0.011 | 0.00000729 | -0.007 | 0.045 | 0.870 | 20.704 |
|  |  |  | rs12760724 | A | C | -0.048 | 0.011 | 0.00000727 | 0.066 | 0.046 | 0.146 | 20.020 |
|  |  |  | rs13005175 | A | G | 0.099 | 0.022 | 0.00000837 | -0.034 | 0.105 | 0.743 | 19.984 |
|  |  |  | rs2159863 | A | G | -0.059 | 0.013 | 0.0000037 | -0.041 | 0.056 | 0.458 | 20.006 |
|  |  |  | rs2910921 | T | C | 0.160 | 0.036 | 0.00000842 | -0.217 | 0.127 | 0.089 | 20.347 |
|  |  |  | rs3127230 | C | T | -0.050 | 0.011 | 0.0000062 | -0.002 | 0.047 | 0.959 | 20.969 |
|  |  |  | rs35524804 | T | C | -0.061 | 0.013 | 0.00000245 | 0.077 | 0.052 | 0.138 | 19.850 |
|  |  |  | rs7359994 | T | C | -0.050 | 0.011 | 0.00000536 | 0.017 | 0.044 | 0.694 | 20.691 |
|  |  |  | rs79086868 | T | C | 0.078 | 0.016 | 0.00000301 | 0.001 | 0.071 | 0.991 | 20.008 |
|  |  |  | rs959845 | C | T | -0.049 | 0.011 | 0.00000517 | 0.030 | 0.044 | 0.501 | 20.153 |
|  |  |  | rs9929145 | G | A | -0.126 | 0.025 | 0.000000284 | 0.058 | 0.098 | 0.557 | 20.138 |
|  | Family | Desulfovibrionaceae | rs112381107 | T | C | 0.211 | 0.046 | 0.00000282 | 0.184 | 0.091 | 0.042 | 16.911 |
|  |  |  | rs11599763 | C | T | -0.056 | 0.012 | 0.0000025 | -0.007 | 0.044 | 0.883 | 21.942 |
|  |  |  | rs16851319 | C | G | -0.073 | 0.015 | 0.0000024 | -0.028 | 0.057 | 0.629 | 20.291 |
|  |  |  | rs17791387 | G | A | -0.073 | 0.015 | 0.0000021 | -0.029 | 0.072 | 0.689 | 22.385 |
|  |  |  | rs2692012 | G | A | 0.114 | 0.025 | 0.00000156 | 0.012 | 0.095 | 0.897 | 21.154 |
|  |  |  | rs2838334 | A | G | 0.057 | 0.012 | 0.00000382 | 0.011 | 0.045 | 0.816 | 21.399 |
|  |  |  | rs3935584 | T | C | -0.053 | 0.012 | 0.00000678 | -0.060 | 0.043 | 0.160 | 23.553 |
|  |  |  | rs4506934 | T | C | -0.094 | 0.020 | 0.00000316 | -0.093 | 0.067 | 0.164 | 22.311 |
|  |  |  | rs6058181 | T | C | 0.083 | 0.017 | 0.00000027 | 0.047 | 0.058 | 0.418 | 21.139 |
|  |  |  | rs7164160 | T | A | -0.057 | 0.013 | 0.00000734 | 0.051 | 0.056 | 0.359 | 19.685 |
|  |  |  | rs72647048 | C | T | -0.077 | 0.017 | 0.00000961 | 0.035 | 0.067 | 0.602 | 20.632 |
|  |  |  | rs9928243 | A | C | -0.054 | 0.012 | 0.00000448 | -0.056 | 0.043 | 0.195 | 20.304 |
|  | Genus | Senegalimassilia | rs10036909 | T | C | 0.186 | 0.040 | 0.00000805 | -0.142 | 0.110 | 0.195 | 22.572 |
|  |  |  | rs11787826 | A | C | 0.081 | 0.017 | 0.00000263 | -0.024 | 0.043 | 0.581 | 21.901 |
|  |  |  | rs13383270 | C | G | 0.077 | 0.017 | 0.00000604 | -0.036 | 0.043 | 0.396 | 21.577 |
|  |  |  | rs1990708 | C | A | -0.110 | 0.025 | 0.00000891 | -0.012 | 0.079 | 0.876 | 22.630 |
|  |  |  | rs2017373 | T | C | 0.078 | 0.018 | 0.0000095 | -0.041 | 0.045 | 0.359 | 21.408 |
|  |  |  | rs57512504 | A | T | 0.082 | 0.017 | 0.00000203 | -0.023 | 0.043 | 0.601 | 20.556 |
|  |  |  | rs7225245 | A | G | 0.079 | 0.017 | 0.00000418 | -0.080 | 0.043 | 0.064 | 19.566 |
|  |  |  | rs72887800 | A | T | -0.082 | 0.018 | 0.00000242 | 0.041 | 0.044 | 0.350 | 19.562 |
|  | Genus | Ruminococcaceae_NK4A214_group | rs11241747 | T | C | 0.053 | 0.012 | 0.00000659 | -0.076 | 0.047 | 0.104 | 26.490 |
|  |  |  | rs114244418 | G | C | -0.175 | 0.037 | 0.00000359 | -0.148 | 0.109 | 0.176 | 24.309 |
|  |  |  | rs11586410 | A | G | -0.086 | 0.017 | 0.000000366 | 0.044 | 0.060 | 0.463 | 25.811 |
|  |  |  | rs12642039 | C | T | -0.055 | 0.012 | 0.00000343 | 0.078 | 0.045 | 0.081 | 20.078 |
|  |  |  | rs12731 | G | A | -0.053 | 0.012 | 0.00000487 | 0.038 | 0.044 | 0.387 | 22.099 |
|  |  |  | rs13087692 | G | T | 0.057 | 0.013 | 0.00000869 | 0.006 | 0.047 | 0.905 | 22.033 |
|  |  |  | rs136761 | A | G | -0.059 | 0.012 | 0.000000815 | -0.030 | 0.045 | 0.503 | 23.069 |
|  |  |  | rs147475196 | G | A | -0.134 | 0.030 | 0.00000472 | 0.067 | 0.070 | 0.343 | 21.033 |
|  |  |  | rs34576931 | C | G | -0.087 | 0.019 | 0.00000472 | -0.044 | 0.084 | 0.600 | 21.262 |
|  |  |  | rs35559912 | C | T | -0.093 | 0.020 | 0.00000489 | 0.009 | 0.066 | 0.896 | 20.531 |
|  |  |  | rs4814689 | T | C | -0.108 | 0.023 | 0.00000455 | 0.097 | 0.105 | 0.356 | 19.995 |
|  |  |  | rs5994253 | G | A | -0.081 | 0.016 | 0.000000235 | 0.010 | 0.062 | 0.871 | 21.449 |
|  |  |  | rs62027366 | C | T | 0.062 | 0.014 | 0.00000658 | 0.056 | 0.053 | 0.293 | 20.626 |
|  |  |  | rs6681678 | T | C | -0.100 | 0.024 | 0.00000905 | 0.109 | 0.119 | 0.360 | 20.815 |
|  |  |  | rs73158814 | G | C | -0.109 | 0.023 | 0.0000022 | 0.095 | 0.113 | 0.404 | 17.419 |
|  |  |  | rs7573569 | C | T | 0.108 | 0.023 | 0.00000323 | -0.128 | 0.089 | 0.151 | 19.778 |
|  | Genus | Prevotella_7 | rs118038478 | G | A | 0.206 | 0.047 | 0.00000785 | 0.041 | 0.084 | 0.628 | 21.931 |
|  |  |  | rs12124567 | G | A | -0.121 | 0.028 | 0.00000949 | -0.055 | 0.054 | 0.310 | 24.188 |
|  |  |  | rs12195431 | C | T | 0.197 | 0.044 | 0.00000873 | 0.105 | 0.073 | 0.153 | 23.320 |
|  |  |  | rs16937247 | C | G | 0.146 | 0.035 | 0.00000964 | 0.017 | 0.054 | 0.759 | 22.118 |
|  |  |  | rs2240542 | T | C | 0.121 | 0.026 | 0.00000484 | 0.064 | 0.049 | 0.185 | 21.300 |
|  |  |  | rs2918132 | T | C | -0.115 | 0.025 | 0.00000642 | 0.027 | 0.044 | 0.543 | 20.215 |
|  |  |  | rs430270 | C | A | 0.139 | 0.030 | 0.00000287 | 0.069 | 0.055 | 0.210 | 17.263 |
|  |  |  | rs57404562 | A | C | 0.155 | 0.032 | 0.000000622 | 0.001 | 0.063 | 0.991 | 19.227 |
|  |  |  | rs79263163 | C | A | -0.144 | 0.032 | 0.00000751 | -0.074 | 0.053 | 0.162 | 19.436 |
|  |  |  | rs9426434 | C | T | -0.124 | 0.028 | 0.00000972 | -0.049 | 0.045 | 0.283 | 19.730 |
|  |  |  | rs9608249 | G | A | -0.158 | 0.034 | 0.00000207 | 0.016 | 0.067 | 0.816 | 19.701 |
|  |  |  | rs9959718 | A | G | 0.133 | 0.028 | 0.0000019 | -0.039 | 0.053 | 0.457 | 20.879 |
|  | Genus | Faecalibacterium | rs10927394 | T | G | -0.232 | 0.051 | 0.00000702 | -0.167 | 0.155 | 0.282 | 33.449 |
|  |  |  | rs114946999 | T | C | -0.086 | 0.019 | 0.0000057 | -0.033 | 0.065 | 0.614 | 23.695 |
|  |  |  | rs11776390 | C | T | -0.078 | 0.017 | 0.0000064 | -0.051 | 0.087 | 0.558 | 23.193 |
|  |  |  | rs12320842 | G | C | 0.095 | 0.016 | 7.57E-09 | 0.082 | 0.063 | 0.191 | 23.890 |
|  |  |  | rs1271565 | T | C | -0.058 | 0.012 | 0.0000013 | 0.052 | 0.049 | 0.287 | 20.024 |
|  |  |  | rs12753492 | C | A | 0.064 | 0.015 | 0.0000088 | 0.025 | 0.068 | 0.711 | 21.303 |
|  |  |  | rs2835874 | C | T | -0.087 | 0.020 | 0.00000754 | -0.147 | 0.113 | 0.194 | 20.538 |
|  |  |  | rs28376661 | G | C | 0.050 | 0.011 | 0.00000366 | 0.046 | 0.047 | 0.328 | 20.646 |
|  |  |  | rs61875484 | G | C | 0.082 | 0.018 | 0.00000918 | -0.041 | 0.073 | 0.571 | 20.321 |
|  |  |  | rs6910935 | G | A | 0.135 | 0.028 | 0.00000138 | -0.009 | 0.089 | 0.924 | 19.826 |
|  |  |  | rs75499067 | T | C | 0.228 | 0.047 | 0.00000176 | 0.129 | 0.082 | 0.117 | 18.293 |
|  |  |  | rs79656633 | C | T | 0.146 | 0.032 | 0.00000814 | 0.087 | 0.072 | 0.226 | 19.437 |
|  |  |  | rs9536330 | C | T | -0.048 | 0.011 | 0.00000533 | 0.004 | 0.043 | 0.920 | 20.791 |

# Supplementary Table 4 Mendelian randomization analysis results between gut microbiota and thyroid nodules

| **outcome** | **Taxa** | **Taxa/gut microbiota** | **nSNP** | **Method of MR** | **p-value** | **OR** | **CI_lower** | **CI_upper** | **OR (95%CI)** | **p_FDR_** | **SE** |
| --- | --- | --- | --- | --- | --- | --- | --- | --- | --- | --- | --- |
| Thyroid nodule | Class | Class / Deltaproteobacteria | 13 | IVW | 0.036 | 0.646249 | 0.429919 | 0.971433 | 0.65 (0.43-0.97) | 0.871 | 0.208 |
|  |  |  |  | MR Egger | 0.135 | 0.411226 | 0.139763 | 1.209955 | 0.41（0.14-1.21） |  | 0.551 |
|  |  |  |  | Weighted median | 0.101 | 0.629578 | 0.362351 | 1.09388 | 0.63（0.36-1.09） |  | 0.282 |
|  |  |  |  | Simple mode | 0.532 | 0.736033 | 0.289428 | 1.871775 | 0.74 (0.29-1.87) |  | 0.476 |
|  |  |  |  | Weighted mode | 0.083 | 0.455027 | 0.200962 | 1.030295 | 0.46 (0.20-1.03) |  | 0.417 |
|  | Order | Order / Desulfovibrionales | 12 | IVW | 0.028 | 0.626908 | 0.412951 | 0.951718 | 0.63 (0.41-0.95) | 0.871 | 0.213 |
|  |  |  |  | MR Egger | 0.102 | 0.380574 | 0.132818 | 1.090491 | 0.38 (0.13-1.09) |  | 0.537 |
|  |  |  |  | Weighted median | 0.054 | 0.574859 | 0.327604 | 1.008727 | 0.57 (0.33-1.01) |  | 0.287 |
|  |  |  |  | Simple mode | 0.502 | 0.718324 | 0.282338 | 1.827557 | 0.72 (0.28-1.83) |  | 0.476 |
|  |  |  |  | Weighted mode | 0.06 | 0.44518 | 0.208795 | 0.949187 | 0.45 (0.21-0.95) |  | 0.386 |
|  | Family | Family / Lachnospiraceae | 16 | Inverse variance weighted | 0.045 | 0.638321 | 0.411374 | 0.99047 | 0.64 (0.41-0.99) | 0.871 | 0.224 |
|  |  |  |  | MR Egger | 0.272 | 0.410714 | 0.089268 | 1.889657 | 0.41 (0.09-1.89) |  | 0.779 |
|  |  |  |  | Weighted median | 0.211 | 0.686583 | 0.380776 | 1.23799 | 0.69 (0.38-1.24) |  | 0.301 |
|  |  |  |  | Simple mode | 0.536 | 0.714438 | 0.252286 | 2.02319 | 0.71 (0.25-2.02) |  | 0.531 |
|  |  |  |  | Weighted mode | 0.555 | 0.720368 | 0.248321 | 2.08976 | 0.72 (0.25-2.09) |  | 0.543 |
|  | Family | Family/ Desulfovibrionaceae | 10 | Inverse variance weighted | 0.008 | 0.551145 | 0.353946 | 0.85821 | 0.55 (0.35-0.86) | 0.871 | 0.226 |
|  |  |  |  | MR Egger | 0.185 | 0.451926 | 0.154668 | 1.320484 | 0.45 (0.15-1.32) |  | 0.547 |
|  |  |  |  | Weighted median | 0.034 | 0.511964 | 0.275959 | 0.949805 | 0.51 (0.28-0.95) |  | 0.315 |
|  |  |  |  | Simple mode | 0.521 | 0.738117 | 0.302577 | 1.800589 | 0.74 (0.30-1.80) |  | 0.455 |
|  |  |  |  | Weighted mode | 0.063 | 0.419137 | 0.187562 | 0.936627 | 0.42 (0.19-0.94) |  | 0.41 |
|  | Genus | Genus / Senegalimassilia | 5 | Inverse variance weighted | 0.037 | 1.723332 | 1.033875 | 2.872563 | 1.72 (1.03-2.87) | 0.871 | 0.261 |
|  |  |  |  | MR Egger | 0.634 | 1.707225 | 0.234311 | 12.43909 | 1.71 (0.23-12.44) |  | 1.013 |
|  |  |  |  | Weighted median | 0.117 | 1.695211 | 0.87647 | 3.278765 | 1.70 (0.88-3.28) |  | 0.337 |
|  |  |  |  | Simple mode | 0.221 | 1.847364 | 0.804902 | 4.23996 | 1.85 (0.80-4.24) |  | 0.424 |
|  |  |  |  | Weighted mode | 0.294 | 1.696764 | 0.718569 | 4.006586 | 1.70 (0.72-4.01) |  | 0.438 |
|  |  | Genus / Ruminococcaceae_NK4A214_group | 13 | Inverse variance weighted | 0.04 | 1.557502 | 1.020768 | 2.376458 | 1.56 (1.02-2.38) |  | 0.216 |
|  |  |  |  | MR Egger | 0.39 | 1.891935 | 0.468513 | 7.639956 | 1.89 (0.47-7.64) |  | 0.712 |
|  |  |  |  | Weighted median | 0.081 | 1.648695 | 0.940812 | 2.8892 | 1.65 (0.94-2.89) |  | 0.286 |
|  |  |  |  | Simple mode | 0.148 | 2.279741 | 0.801642 | 6.483216 | 2.28 (0.80-6.48) |  | 0.533 |
|  |  |  |  | Weighted mode | 0.294 | 1.54965 | 0.708844 | 3.387792 | 1.55 (0.71-3.39) |  | 0.399 |
|  | Genus | Genus / Prevotella_7 | 11 | Inverse variance weighted | 0.049 | 0.79054 | 0.625837 | 0.998588 | 0.79 (0.63-1.00) | 0.871 | 0.119 |
|  |  |  |  | MR Egger | 0.517 | 0.626513 | 0.161093 | 2.436596 | 0.63 (0.16-2.44) |  | 0.693 |
|  |  |  |  | Weighted median | 0.015 | 0.679813 | 0.498138 | 0.927746 | 0.68 (0.50-0.93) |  | 0.159 |
|  |  |  |  | Simple mode | 0.101 | 0.614346 | 0.361799 | 1.043181 | 0.61 (0.36-1.04) |  | 0.27 |
|  |  |  |  | Weighted mode | 0.101 | 0.614346 | 0.362035 | 1.042499 | 0.61 (0.36-1.04) |  | 0.27 |
|  | Genus | Genus / Faecalibacterium | 10 | Inverse variance weighted | 0.05 | 0.664119 | 0.441425 | 0.99916 | 0.66 (0.44-1.00) | 0.871 | 0.208 |
|  |  |  |  | MR Egger | 0.074 | 0.433245 | 0.195203 | 0.961572 | 0.43 (0.20-0.96) |  | 0.407 |
|  |  |  |  | Weighted median | 0.045 | 0.571441 | 0.331037 | 0.986428 | 0.57 (0.33-0.99) |  | 0.279 |
|  |  |  |  | Simple mode | 0.179 | 0.568038 | 0.265336 | 1.216068 | 0.57 (0.27-1.22) |  | 0.388 |
|  |  |  |  | Weighted mode | 0.089 | 0.560011 | 0.308781 | 1.015645 | 0.56 (0.31-1.02) |  | 0.304 |

# Supplementary Table 5 Heterogeneity and Pleiotropy tests for MR analysis of gut microbiota and thyroid nodules

| **Outcome** | **Taxa/Gut microbiota** | **Egger intercept analysis** | | | **Cochran's Q test** | | | |
| --- | --- | --- | --- | --- | --- | --- | --- | --- |
|  |  |  |  |  | **MR-Egger** | | **IVW** | |
|  |  | **egger-intercept** | **SE** | ***P*-value** | **Q-df** | ***P*-value** | **Q-df** | ***P*** |
| Thyroid nodules | Class / Deltaproteobacteria | -0.036 | 0.040 | 0.394 | 11 | 0.797 | 12 | 0.799 |
|  | Order / Desulfovibrionales | -0.040 | 0.039 | 0.335 | 10 | 0.814 | 11 | 0.796 |
|  | Family / Lachnospiraceae | 0.028 | 0.047 | 0.564 | 14 | 0.845 | 15 | 0.871 |
|  | Family/ Desulfovibrionaceae | -0.017 | 0.042 | 0.701 | 8 | 0.876 | 9 | 0.915 |
|  | Genus / Senegalimassilia | -0.001 | 0.091 | 0.993 | 3 | 0.586 | 4 | 0.747 |
|  | Genus / Ruminococcaceae_NK4A214_group | 0.015 | 0.052 | 0.780 | 11 | 0.628 | 12 | 0.701 |
|  | Genus / Prevotella_7 | -0.033 | 0.097 | 0.741 | 9 | 0.666 | 10 | 0.741 |
|  | Genus / Faecalibacterium | -0.052 | 0.042 | 0.256 | 8 | 0.914 | 9 | 0.852 |

# Supplementary Table 6 MR-PRESSO analysis for the association between gut microbiota and thyroid nodules

| **Outcome** | **Taxa/Gut microbiota** | **Causal Estimate** | **SD** | **T-stat** | ***P*-value** | **RSS_obs_** | **Global test *P*-value** | **NO. outliers** |
| --- | --- | --- | --- | --- | --- | --- | --- | --- |
| Thyroid nodules | Class / Deltaproteobacteria | 0.437 | 0.168 | 2.601 | 0.023 | 9.421 | 0.798 | NA |
|  | Order / Desulfovibrionales | 0.467 | 0.170 | 2.740 | 0.019 | 8.571 | 0.797 | NA |
|  | Family / Lachnospiraceae | -0.449 | 0.175 | -2.568 | 0.021 | 10.405 | 0.868 | NA |
|  | Family/ Desulfovibrionaceae | 0.596 | 0.149 | 3.986 | 0.003 | 4.918 | 0.919 | NA |
|  | Genus / Senegalimassilia | -0.544 | 0.181 | -3.001 | 0.040 | 2.942 | 0.774 | NA |
|  | Genus / Ruminococcaceae_NK4A214_group | -0.443 | 0.187 | -2.371 | 0.035 | 10.382 | 0.729 | NA |
|  | Genus / Prevotella_7 | 0.235 | 0.099 | 2.385 | 0.038 | 8.296 | 0.744 | NA |
|  | Genus / Faecalibacterium | 0.409 | 0.152 | 2.691 | 0.025 | 5.680 | 0.877 | NA |

# Supplementary Table 7 The Leave-one-out analysis for IVW MR of gut microbiota on thyroid nodules

| **Outcome** | **Taxa/Gut microbiota** | **SNP** | **Beta** | **SE** | ***P-*value** |
| --- | --- | --- | --- | --- | --- |
| Thyroid nodules | Class / Deltaproteobacteria | rs1035691 | 0.453 | 0.215 | 0.035 |
|  |  | rs112381107 | 0.305 | 0.236 | 0.197 |
|  |  | rs11599763 | 0.459 | 0.215 | 0.033 |
|  |  | rs17084793 | 0.499 | 0.214 | 0.020 |
|  |  | rs17791387 | 0.439 | 0.213 | 0.039 |
|  |  | rs2692012 | 0.457 | 0.214 | 0.033 |
|  |  | rs2838334 | 0.455 | 0.215 | 0.035 |
|  |  | rs3935584 | 0.387 | 0.215 | 0.071 |
|  |  | rs4506934 | 0.385 | 0.217 | 0.077 |
|  |  | rs55744759 | 0.491 | 0.214 | 0.022 |
|  |  | rs6058181 | 0.424 | 0.218 | 0.052 |
|  |  | rs62020470 | 0.502 | 0.213 | 0.018 |
|  |  | rs9928243 | 0.393 | 0.215 | 0.068 |
|  |  | All | 0.437 | 0.208 | 0.036 |
|  | Order / Desulfovibrionales | rs112381107 | 0.335 | 0.245 | 0.172 |
|  |  | rs11599763 | 0.494 | 0.221 | 0.025 |
|  |  | rs17791387 | 0.470 | 0.218 | 0.031 |
|  |  | rs186073 | 0.521 | 0.220 | 0.018 |
|  |  | rs2692012 | 0.491 | 0.220 | 0.026 |
|  |  | rs2838334 | 0.489 | 0.221 | 0.027 |
|  |  | rs3935584 | 0.417 | 0.221 | 0.058 |
|  |  | rs4506934 | 0.415 | 0.224 | 0.063 |
|  |  | rs6058181 | 0.457 | 0.224 | 0.041 |
|  |  | rs62020470 | 0.535 | 0.218 | 0.014 |
|  |  | rs72647048 | 0.526 | 0.220 | 0.017 |
|  |  | rs9928243 | 0.423 | 0.221 | 0.056 |
|  |  | All | 0.467 | 0.213 | 0.028 |
|  | Family / Lachnospiraceae | rs10402491 | -0.452 | 0.232 | 0.052 |
|  |  | rs11139361 | -0.373 | 0.231 | 0.106 |
|  |  | rs112040820 | -0.514 | 0.232 | 0.026 |
|  |  | rs11841382 | -0.440 | 0.229 | 0.055 |
|  |  | rs11979110 | -0.508 | 0.232 | 0.029 |
|  |  | rs1205443 | -0.469 | 0.231 | 0.043 |
|  |  | rs12760724 | -0.394 | 0.231 | 0.088 |
|  |  | rs13005175 | -0.454 | 0.229 | 0.048 |
|  |  | rs2159863 | -0.517 | 0.231 | 0.025 |
|  |  | rs2910921 | -0.371 | 0.234 | 0.112 |
|  |  | rs3127230 | -0.480 | 0.231 | 0.038 |
|  |  | rs35524804 | -0.388 | 0.232 | 0.094 |
|  |  | rs7359994 | -0.457 | 0.232 | 0.049 |
|  |  | rs79086868 | -0.478 | 0.231 | 0.039 |
|  |  | rs959845 | -0.439 | 0.232 | 0.058 |
|  |  | rs9929145 | -0.448 | 0.234 | 0.056 |
|  |  | All | -0.449 | 0.224 | 0.045 |
|  | Family/ Desulfovibrionaceae | rs112381107 | 0.490 | 0.266 | 0.065 |
|  |  | rs11599763 | 0.638 | 0.236 | 0.007 |
|  |  | rs17791387 | 0.607 | 0.232 | 0.009 |
|  |  | rs2692012 | 0.635 | 0.235 | 0.007 |
|  |  | rs2838334 | 0.633 | 0.236 | 0.007 |
|  |  | rs3935584 | 0.550 | 0.235 | 0.019 |
|  |  | rs4506934 | 0.552 | 0.238 | 0.021 |
|  |  | rs6058181 | 0.600 | 0.239 | 0.012 |
|  |  | rs72647048 | 0.671 | 0.234 | 0.004 |
|  |  | rs9928243 | 0.558 | 0.236 | 0.018 |
|  |  | All | 0.596 | 0.226 | 0.008 |
|  | Genus / Senegalimassilia | rs10036909 | -0.491 | 0.290 | 0.091 |
|  |  | rs11787826 | -0.623 | 0.299 | 0.037 |
|  |  | rs1990708 | -0.643 | 0.280 | 0.021 |
|  |  | rs2017373 | -0.550 | 0.293 | 0.061 |
|  |  | rs7225245 | -0.406 | 0.297 | 0.171 |
|  |  | All | -0.544 | 0.261 | 0.037 |
|  | Genus / Ruminococcaceae_NK4A214_group | rs11241747 | -0.380 | 0.222 | 0.088 |
|  |  | rs11586410 | -0.436 | 0.227 | 0.055 |
|  |  | rs12642039 | -0.369 | 0.224 | 0.099 |
|  |  | rs12731 | -0.423 | 0.223 | 0.058 |
|  |  | rs13087692 | -0.484 | 0.224 | 0.030 |
|  |  | rs136761 | -0.527 | 0.225 | 0.019 |
|  |  | rs147475196 | -0.432 | 0.236 | 0.068 |
|  |  | rs35559912 | -0.479 | 0.226 | 0.034 |
|  |  | rs4814689 | -0.420 | 0.221 | 0.058 |
|  |  | rs5994253 | -0.471 | 0.225 | 0.036 |
|  |  | rs62027366 | -0.533 | 0.223 | 0.017 |
|  |  | rs6681678 | -0.421 | 0.219 | 0.055 |
|  |  | rs7573569 | -0.389 | 0.223 | 0.082 |
|  |  | All | -0.443 | 0.216 | 0.040 |
|  | Genus / Prevotella_7 | rs118038478 | 0.239 | 0.125 | 0.056 |
|  |  | rs12124567 | 0.218 | 0.124 | 0.078 |
|  |  | rs12195431 | 0.201 | 0.126 | 0.110 |
|  |  | rs2240542 | 0.206 | 0.125 | 0.098 |
|  |  | rs2918132 | 0.285 | 0.125 | 0.023 |
|  |  | rs430270 | 0.209 | 0.125 | 0.095 |
|  |  | rs57404562 | 0.257 | 0.125 | 0.040 |
|  |  | rs79263163 | 0.202 | 0.126 | 0.108 |
|  |  | rs9426434 | 0.216 | 0.126 | 0.086 |
|  |  | rs9608249 | 0.264 | 0.124 | 0.034 |
|  |  | rs9959718 | 0.287 | 0.125 | 0.021 |
|  |  | All | 0.235 | 0.119 | 0.049 |
|  | Genus / Faecalibacterium | rs10927394 | 0.376 | 0.219 | 0.087 |
|  |  | rs114946999 | 0.412 | 0.217 | 0.058 |
|  |  | rs11776390 | 0.400 | 0.212 | 0.059 |
|  |  | rs1271565 | 0.494 | 0.215 | 0.022 |
|  |  | rs12753492 | 0.410 | 0.213 | 0.054 |
|  |  | rs2835874 | 0.376 | 0.211 | 0.075 |
|  |  | rs6910935 | 0.462 | 0.220 | 0.036 |
|  |  | rs75499067 | 0.331 | 0.255 | 0.194 |
|  |  | rs79656633 | 0.368 | 0.230 | 0.109 |
|  |  | rs9536330 | 0.438 | 0.214 | 0.041 |
|  |  | All | 0.409 | 0.208 | 0.050 |

# Supplementary Table 8 The SNPs included according to selection criteria as instrumental variables for thyroid nodules on gut microbiota

| **Exposure** | **Taxa/Gut microbiota** | **SNP** | **Effect_allele** | **Other_allele** | **Thyroid nodule/Exposure** | | | **Gut micriobiota/Outcome** | | |
| --- | --- | --- | --- | --- | --- | --- | --- | --- | --- | --- |
|  |  |  |  |  | **Beta** | **SE** | ***P-*value** | **Beta** | **SE** | ***P-*value** |
| Thyroid nodules | Class / Deltaproteobacteria | rs139990698 | T | C | 0.371 | 0.079 | 0.000 | 0.371 | 0.079 | 0.000 |
|  |  | rs2251739 | T | A | 0.373 | 0.079 | 0.000 | 0.373 | 0.079 | 0.000 |
|  |  | rs4338740 | C | T | 0.217 | 0.046 | 0.000 | 0.217 | 0.046 | 0.000 |
|  |  | rs4812597 | G | A | 0.199 | 0.043 | 0.000 | 0.199 | 0.043 | 0.000 |
|  |  | rs7192598 | C | T | 0.222 | 0.047 | 0.000 | 0.222 | 0.047 | 0.000 |
|  |  | rs73159816 | A | G | 0.296 | 0.065 | 0.000 | 0.296 | 0.065 | 0.000 |
|  | Order / Desulfovibrionales | rs139990698 | T | C | 0.371 | 0.079 | 0.000 | 0.371 | 0.079 | 0.000 |
|  |  | rs2251739 | T | A | 0.373 | 0.079 | 0.000 | 0.373 | 0.079 | 0.000 |
|  |  | rs4338740 | C | T | 0.217 | 0.046 | 0.000 | 0.217 | 0.046 | 0.000 |
|  |  | rs4812597 | G | A | 0.199 | 0.043 | 0.000 | 0.199 | 0.043 | 0.000 |
|  |  | rs7192598 | C | T | 0.222 | 0.047 | 0.000 | 0.222 | 0.047 | 0.000 |
|  |  | rs73159816 | A | G | 0.296 | 0.065 | 0.000 | 0.296 | 0.065 | 0.000 |
|  | Family / Lachnospiraceae | rs139990698 | T | C | 0.371 | 0.079 | 0.000 | 0.371 | 0.079 | 0.000 |
|  |  | rs2251739 | T | A | 0.373 | 0.079 | 0.000 | 0.373 | 0.079 | 0.000 |
|  |  | rs4338740 | C | T | 0.217 | 0.046 | 0.000 | 0.217 | 0.046 | 0.000 |
|  |  | rs4812597 | G | A | 0.199 | 0.043 | 0.000 | 0.199 | 0.043 | 0.000 |
|  |  | rs7192598 | C | T | 0.222 | 0.047 | 0.000 | 0.222 | 0.047 | 0.000 |
|  |  | rs73159816 | A | G | 0.296 | 0.065 | 0.000 | 0.296 | 0.065 | 0.000 |
|  | Family/ Desulfovibrionaceae | rs139990698 | T | C | 0.371 | 0.079 | 0.000 | 0.371 | 0.079 | 0.000 |
|  |  | rs2251739 | T | A | 0.373 | 0.079 | 0.000 | 0.373 | 0.079 | 0.000 |
|  |  | rs4338740 | C | T | 0.217 | 0.046 | 0.000 | 0.217 | 0.046 | 0.000 |
|  |  | rs4812597 | G | A | 0.199 | 0.043 | 0.000 | 0.199 | 0.043 | 0.000 |
|  |  | rs7192598 | C | T | 0.222 | 0.047 | 0.000 | 0.222 | 0.047 | 0.000 |
|  |  | rs73159816 | A | G | 0.296 | 0.065 | 0.000 | 0.296 | 0.065 | 0.000 |
|  | Genus / Senegalimassilia | rs139990698 | T | C | 0.371 | 0.079 | 0.000 | 0.371 | 0.079 | 0.000 |
|  |  | rs2251739 | T | A | 0.373 | 0.079 | 0.000 | 0.373 | 0.079 | 0.000 |
|  |  | rs4338740 | C | T | 0.217 | 0.046 | 0.000 | 0.217 | 0.046 | 0.000 |
|  |  | rs4812597 | G | A | 0.199 | 0.043 | 0.000 | 0.199 | 0.043 | 0.000 |
|  |  | rs7192598 | C | T | 0.222 | 0.047 | 0.000 | 0.222 | 0.047 | 0.000 |
|  |  | rs73159816 | A | G | 0.296 | 0.065 | 0.000 | 0.296 | 0.065 | 0.000 |
|  | Genus / Ruminococcaceae_NK4A214_group | rs139990698 | T | C | 0.371 | 0.079 | 0.000 | 0.371 | 0.079 | 0.000 |
|  |  | rs2251739 | T | A | 0.373 | 0.079 | 0.000 | 0.373 | 0.079 | 0.000 |
|  |  | rs4338740 | C | T | 0.217 | 0.046 | 0.000 | 0.217 | 0.046 | 0.000 |
|  |  | rs4812597 | G | A | 0.199 | 0.043 | 0.000 | 0.199 | 0.043 | 0.000 |
|  |  | rs7192598 | C | T | 0.222 | 0.047 | 0.000 | 0.222 | 0.047 | 0.000 |
|  |  | rs73159816 | A | G | 0.296 | 0.065 | 0.000 | 0.296 | 0.065 | 0.000 |
|  | Genus / Prevotella_7 | rs139990698 | T | C | 0.371 | 0.079 | 0.000 | 0.371 | 0.079 | 0.000 |
|  |  | rs2251739 | T | A | 0.373 | 0.079 | 0.000 | 0.373 | 0.079 | 0.000 |
|  |  | rs4338740 | C | T | 0.217 | 0.046 | 0.000 | 0.217 | 0.046 | 0.000 |
|  |  | rs4812597 | G | A | 0.199 | 0.043 | 0.000 | 0.199 | 0.043 | 0.000 |
|  |  | rs7192598 | C | T | 0.222 | 0.047 | 0.000 | 0.222 | 0.047 | 0.000 |
|  |  | rs73159816 | A | G | 0.296 | 0.065 | 0.000 | 0.296 | 0.065 | 0.000 |
|  | Genus / Faecalibacterium | rs139990698 | T | C | 0.371 | 0.079 | 0.000 | 0.371 | 0.079 | 0.000 |
|  |  | rs2251739 | T | A | 0.373 | 0.079 | 0.000 | 0.373 | 0.079 | 0.000 |
|  |  | rs4338740 | C | T | 0.217 | 0.046 | 0.000 | 0.217 | 0.046 | 0.000 |
|  |  | rs4812597 | G | A | 0.199 | 0.043 | 0.000 | 0.199 | 0.043 | 0.000 |
|  |  | rs7192598 | C | T | 0.222 | 0.047 | 0.000 | 0.222 | 0.047 | 0.000 |
|  |  | rs73159816 | A | G | 0.296 | 0.065 | 0.000 | 0.296 | 0.065 | 0.000 |

# Supplementary Table 9 Causal estiamte from thyroid nodules exposure to identified microbiota outcomes in the reverse MR analysis

| **Disease** | **Taxa/gut microbiota** | **nSNP** | **Method of MR** | **Beta** | **SE** | ***P*-value** | **OR（95%CI）** |
| --- | --- | --- | --- | --- | --- | --- | --- |
| Thyroid nodules | Class / Deltaproteobacteria | 5 | Inverse variance weighted | -0.002 | 0.023 | 0.946 | 1.00（0.95-1.05） |
|  |  |  | MR Egger | -0.033 | 0.097 | 0.790 | 0.97（0.77-1.21） |
|  |  |  | Weighted median | -0.022 | 0.031 | 0.473 | 0.98（0.92-1.04） |
|  | Order / Desulfovibrionales | 5 | Inverse variance weighted | -0.001 | 0.025 | 0.982 | 1.00（0.95-1.05） |
|  |  |  | MR Egger | -0.022 | 0.115 | 0.860 | 0.98（0.78-1.23） |
|  |  |  | Weighted median | -0.025 | 0.032 | 0.435 | 0.97（0.91-1.04） |
|  | Family / Lachnospiraceae | 5 | Inverse variance weighted | -0.005 | 0.023 | 0.816 | 0.99（0.95-1.04） |
|  |  |  | MR Egger | -0.142 | 0.105 | 0.268 | 0.87（0.71-1.07） |
|  |  |  | Weighted median | 0.008 | 0.031 | 0.790 | 1.01（0.95-1.07） |
|  | Family/ Desulfovibrionaceae | 5 | Inverse variance weighted | -0.002 | 0.025 | 0.945 | 1.00（0.95-1.05） |
|  |  |  | MR Egger | -0.023 | 0.115 | 0.854 | 0.98(0.78-1.22) |
|  |  |  | Weighted median | -0.026 | 0.032 | 0.410 | 0.97（0.92-1.04） |
|  | Genus / Senegalimassilia | 5 | Inverse variance weighted | -0.048 | 0.052 | 0.354 | 0.95（0.86-1.06） |
|  |  |  | MR Egger | -0.036 | 0.277 | 0.904 | 0.96（0.56-1.66） |
|  |  |  | Weighted median | 0.023 | 0.054 | 0.673 | 1.02（0.92-1.14） |
|  | Genus / Ruminococcaceae_NK4A214_group | 5 | Inverse variance weighted | 0.008 | 0.025 | 0.747 | 1.01（0.96-1.06） |
|  |  |  | MR Egger | 0.080 | 0.115 | 0.533 | 1.08（0.87-1.36） |
|  |  |  | Weighted median | 0.023 | 0.031 | 0.464 | 1.02（0.96-1.09） |
|  | Genus / Prevotella_7 | 5 | Inverse variance weighted | 0.087 | 0.052 | 0.094 | 1.09（0.99-1.21） |
|  |  |  | MR Egger | 0.068 | 0.245 | 0.800 | 1.07（0.66-1.73） |
|  |  |  | Weighted median | 0.041 | 0.071 | 0.570 | 1.04（0.91-1.20） |
|  | Genus / Faecalibacterium | 5 | Inverse variance weighted | -0.023 | 0.029 | 0.425 | 0.98（0.92-1.03） |
|  |  |  | MR Egger | 0.077 | 0.144 | 0.631 | 1.08（0.81-1.43） |
|  |  |  | Weighted median | -0.023 | 0.034 | 0.501 | 0.98（0.92-1.04） |

# Supplementary Table 10 Heterogeneity and Pleiotropy tests for reverse MR analysis of thyroid nodules on gut microbiota

| **Exposure** | **Taxa/Gut microbiota** | **Egger intercept analysis** | | | **Cochran's Q test** | | | | **MR_PRESSO** | |
| --- | --- | --- | --- | --- | --- | --- | --- | --- | --- | --- |
|  |  |  |  |  | **MR-Egger** | | **IVW** | |  |  |
|  |  | **egger-intercept** | **SE** | ***P*-value** | **Q_df** | ***P*** | **Q_df** | ***P*** | **Global test *P*-value** | **NO. outliers** |
| Thyroid nodules | Class / Deltaproteobacteria | 0.008 | 0.028 | 0.796 | 3 | 0.531 | 4 | 0.683 | 0.688 | NA |
|  | Order / Desulfovibrionales | 0.005 | 0.028 | 0.861 | 3 | 0.535 | 4 | 0.695 | 0.708 | NA |
|  | Family / Lachnospiraceae | 0.035 | 0.026 | 0.274 | 3 | 0.480 | 4 | 0.372 | 0.399 | NA |
|  | Family/ Desulfovibrionaceae | 0.005 | 0.028 | 0.862 | 3 | 0.515 | 4 | 0.677 | 0.688 | NA |
|  | Genus / Senegalimassilia | -0.003 | 0.069 | 0.967 | 3 | 0.040 | 4 | 0.081 | 0.129 | NA |
|  | Genus / Ruminococcaceae_NK4A214_group | -0.018 | 0.028 | 0.563 | 3 | 0.684 | 4 | 0.751 | 0.776 | NA |
|  | Genus / Prevotella_7 | 0.005 | 0.060 | 0.941 | 3 | 0.369 | 4 | 0.531 | 0.551 | NA |
|  | Genus / Faecalibacterium | -0.025 | 0.036 | 0.528 | 3 | 0.132 | 4 | 0.161 | 0.200 | NA |

# Supplementary Table 11 Leave-one-out analysis for IVW MR of thyroid nodules on gut microbiota

| **Exposure** | **Taxa/Gut microbiota** | **SNP** | **Beta** | **SE** | ***P*-value** |
| --- | --- | --- | --- | --- | --- |
| Thyroid nodules | Class / Deltaproteobacteria | rs139990698 | 0.005 | 0.028 | 0.847 |
|  |  | rs4338740 | 0.002 | 0.027 | 0.932 |
|  |  | rs4812597 | -0.014 | 0.028 | 0.614 |
|  |  | rs7192598 | 0.010 | 0.028 | 0.730 |
|  |  | rs73159816 | -0.012 | 0.028 | 0.677 |
|  |  | All | -0.002 | 0.025 | 0.946 |
|  | Order / Desulfovibrionales | rs139990698 | 0.007 | 0.028 | 0.813 |
|  |  | rs4338740 | 0.004 | 0.027 | 0.874 |
|  |  | rs4812597 | -0.011 | 0.028 | 0.681 |
|  |  | rs7192598 | 0.010 | 0.028 | 0.724 |
|  |  | rs73159816 | -0.012 | 0.028 | 0.662 |
|  |  | All | -0.001 | 0.025 | 0.982 |
|  | Family / Lachnospiraceae | rs139990698 | 0.017 | 0.026 | 0.517 |
|  |  | rs4338740 | -0.002 | 0.029 | 0.951 |
|  |  | rs4812597 | -0.017 | 0.025 | 0.499 |
|  |  | rs7192598 | -0.009 | 0.030 | 0.777 |
|  |  | rs73159816 | -0.016 | 0.028 | 0.568 |
|  |  | All | -0.005 | 0.023 | 0.816 |
|  | Family/ Desulfovibrionaceae | rs139990698 | 0.006 | 0.028 | 0.832 |
|  |  | rs4338740 | 0.003 | 0.027 | 0.911 |
|  |  | rs4812597 | -0.012 | 0.028 | 0.655 |
|  |  | rs7192598 | 0.009 | 0.028 | 0.750 |
|  |  | rs73159816 | -0.014 | 0.028 | 0.615 |
|  |  | All | -0.002 | 0.025 | 0.945 |
|  | Genus / Senegalimassilia | rs139990698 | -0.071 | 0.063 | 0.258 |
|  |  | rs4338740 | -0.070 | 0.059 | 0.234 |
|  |  | rs4812597 | -0.028 | 0.060 | 0.647 |
|  |  | rs7192598 | -0.067 | 0.064 | 0.294 |
|  |  | rs73159816 | -0.006 | 0.044 | 0.889 |
|  |  | All | -0.048 | 0.052 | 0.354 |
|  | Genus / Ruminococcaceae_NK4A214_group | rs139990698 | -0.002 | 0.028 | 0.936 |
|  |  | rs4338740 | 0.005 | 0.027 | 0.848 |
|  |  | rs4812597 | 0.021 | 0.027 | 0.445 |
|  |  | rs7192598 | 0.001 | 0.028 | 0.965 |
|  |  | rs73159816 | 0.014 | 0.028 | 0.607 |
|  |  | All | 0.008 | 0.025 | 0.747 |
|  | Genus / Prevotella_7 | rs139990698 | 0.110 | 0.059 | 0.062 |
|  |  | rs4338740 | 0.095 | 0.057 | 0.099 |
|  |  | rs4812597 | 0.072 | 0.057 | 0.209 |
|  |  | rs7192598 | 0.110 | 0.058 | 0.058 |
|  |  | rs73159816 | 0.048 | 0.058 | 0.411 |
|  |  | All | 0.087 | 0.052 | 0.094 |
|  | Genus / Faecalibacterium | rs139990698 | -0.023 | 0.038 | 0.558 |
|  |  | rs4338740 | -0.008 | 0.030 | 0.786 |
|  |  | rs4812597 | -0.029 | 0.037 | 0.430 |
|  |  | rs7192598 | -0.010 | 0.034 | 0.776 |
|  |  | rs73159816 | -0.048 | 0.026 | 0.061 |
|  |  | All | -0.023 | 0.029 | 0.425 |

# Supplementary Table 12 Multivariable mendelian randomization analysis results between gut micriobiota and thyroid nodules (adjusting five variables separately)

| **Outcome** | **Taxa/gut microbiota** | **Adjusting variables** | **nSNP** | **methods of multivariable MR** | **Beta** | **SE** | ***P*-value** | **OR（95%CI）** |
| --- | --- | --- | --- | --- | --- | --- | --- | --- |
| Thyroid nodules | Class / Deltaproteobacteria | High blood pressure | 338 | Multivariable IVW | 0.066 | 0.067 | 0.323 | 1.07 (0.94-1.22) |
|  | Class / Deltaproteobacteria |  |  | Multivariable Median | 0.040 | 0.096 | 0.675 | 1.04 (0.86-1.26) |
|  | Class / Deltaproteobacteria |  |  | Multivariable Egger | 0.066 | 0.067 | 0.325 | 1.07 (0.94-1.22) |
|  | Class / Deltaproteobacteria | Obesity class 3 | 21 | Multivariable IVW | -0.099 | 0.061 | 0.105 | 0.91 (0.80-1.02) |
|  | Class / Deltaproteobacteria |  |  | Multivariable Median | -0.110 | 0.089 | 0.215 | 0.90 (0.75-1.07) |
|  | Class / Deltaproteobacteria |  |  | Multivariable Egger | -0.098 | 0.061 | 0.109 | 0.91 (0.80-1.02) |
|  | Class / Deltaproteobacteria | Alcohol consumption | 19 | Multivariable IVW | 0.022 | 0.130 | 0.863 | 1.02 (0.79-1.32) |
|  | Class / Deltaproteobacteria |  |  | Multivariable Median | -0.002 | 0.162 | 0.989 | 1.00 (0.73-1.37) |
|  | Class / Deltaproteobacteria |  |  | Multivariable Egger | -0.004 | 0.135 | 0.979 | 1.00 (0.76-1.30) |
|  | Class / Deltaproteobacteria | Type 2 diabetes | 123 | Multivariable IVW | -0.096 | 0.065 | 0.143 | 0.91 (0.80-1.03) |
|  | Class / Deltaproteobacteria |  |  | Multivariable Median | -0.073 | 0.095 | 0.443 | 0.93 (0.77-1.12) |
|  | Class / Deltaproteobacteria |  |  | Multivariable Egger | -0.092 | 0.066 | 0.162 | 0.91 (0.80-1.04) |
|  | Class / Deltaproteobacteria | Ever smoked | 61 | Multivariable IVW | 0.283 | 0.576 | 0.623 | 1.33 (0.43-4.10) |
|  | Class / Deltaproteobacteria |  |  | Multivariable Median | 0.147 | 0.836 | 0.860 | 1.16 (0.22-5.97) |
|  | Class / Deltaproteobacteria |  |  | Multivariable Egger | 0.358 | 0.579 | 0.536 | 1.43 (0.46-4.45) |
|  | Order / Desulfovibrionales | High blood pressure | 337 | Multivariable IVW | 0.067 | 0.149 | 0.315 | 1.07 (0.80-1.43) |
|  | Order / Desulfovibrionales |  |  | Multivariable Median | 0.053 | 0.097 | 0.585 | 1.05 (0.87-1.28) |
|  | Order / Desulfovibrionales |  |  | Multivariable Egger | 0.541 | 0.067 | 0.077 | 1.72 (1.51-1.96) |
|  | Order / Desulfovibrionales | Obesity class 3 | 20 | Multivariable IVW | -0.092 | 0.062 | 0.135 | 0.91 (0.81-1.03) |
|  | Order / Desulfovibrionales |  |  | Multivariable Median | -0.101 | 0.088 | 0.253 | 0.90 (0.76-1.07) |
|  | Order / Desulfovibrionales |  |  | Multivariable Egger | -0.090 | 0.062 | 0.142 | 0.91 (0.81-1.03) |
|  | Order / Desulfovibrionales | Alcohol consumption | 18 | Multivariable IVW | 0.005 | 0.129 | 0.968 | 1.01 (0.78-1.30) |
|  | Order / Desulfovibrionales |  |  | Multivariable Median | -0.002 | 0.164 | 0.992 | 1.00 (0.72-1.38) |
|  | Order / Desulfovibrionales |  |  | Multivariable Egger | -0.021 | 0.134 | 0.873 | 0.98 (0.75-1.27) |
|  | Order / Desulfovibrionales | Type 2 diabetes | 122 | Multivariable IVW | -0.096 | 0.065 | 0.139 | 0.91 (0.80-1.03) |
|  | Order / Desulfovibrionales |  |  | Multivariable Median | -0.074 | 0.094 | 0.431 | 0.93 (0.77-1.12) |
|  | Order / Desulfovibrionales |  |  | Multivariable Egger | -0.089 | 0.066 | 0.173 | 0.91 (0.80-1.04) |
|  | Order / Desulfovibrionales | Ever smoked | 60 | Multivariable IVW | 0.265 | 0.577 | 0.646 | 1.30 (0.42-4.04) |
|  | Order / Desulfovibrionales |  |  | Multivariable Median | 0.145 | 0.825 | 0.860 | 1.16 (0.23-5.82) |
|  | Order / Desulfovibrionales |  |  | Multivariable Egger | 0.341 | 0.579 | 0.556 | 1.41 (0.45-4.38) |
|  | Family / Lachnospiraceae | High blood pressure | 336 | Multivariable IVW | 0.062 | 0.068 | 0.363 | 1.06 (0.93-1.22) |
|  | Family / Lachnospiraceae |  |  | Multivariable Median | 0.076 | 0.096 | 0.431 | 1.08 (0.89-1.30) |
|  | Family / Lachnospiraceae |  |  | Multivariable Egger | 0.063 | 0.068 | 0.353 | 1.07 (0.93-1.22) |
|  | Family / Lachnospiraceae | Obesity class 3 | 23 | Multivariable IVW | -0.096 | 0.062 | 0.124 | 0.91 (0.80-1.03) |
|  | Family / Lachnospiraceae |  |  | Multivariable Median | -0.111 | 0.087 | 0.198 | 0.89 (0.76-1.06) |
|  | Family / Lachnospiraceae |  |  | Multivariable Egger | -0.070 | 0.068 | 0.300 | 0.93 (0.82-1.06) |
|  | Family / Lachnospiraceae | Alcohol consumption | 20 | Multivariable IVW | 0.016 | 0.146 | 0.914 | 1.02 (0.76-1.35) |
|  | Family / Lachnospiraceae |  |  | Multivariable Median | 0.057 | 0.184 | 0.758 | 1.06 (0.74-1.52) |
|  | Family / Lachnospiraceae |  |  | Multivariable Egger | 0.012 | 0.146 | 0.934 | 1.01 (0.76-1.35) |
|  | Family / Lachnospiraceae | Type 2 diabetes | 118 | Multivariable IVW | -0.098 | 0.064 | 0.124 | 0.91 (0.80-1.03) |
|  | Family / Lachnospiraceae |  |  | Multivariable Median | -0.066 | 0.091 | 0.471 | 0.94 (0.78-1.12) |
|  | Family / Lachnospiraceae |  |  | Multivariable Egger | -0.098 | 0.064 | 0.123 | 0.91 (0.80-1.03) |
|  | Family / Lachnospiraceae | Ever smoked | 65 | Multivariable IVW | -0.026 | 0.564 | 0.964 | 0.97 (0.32-2.94) |
|  | Family / Lachnospiraceae |  |  | Multivariable Median | -0.480 | 0.813 | 0.555 | 0.62 (0.13-3.04) |
|  | Family / Lachnospiraceae |  |  | Multivariable Egger | -0.058 | 0.564 | 0.918 | 0.94 (0.31-2.85) |
|  | Family/ Desulfovibrionaceae | High blood pressure | 337 | Multivariable IVW | 0.067 | 0.067 | 0.317 | 1.07 (0.94-1.22) |
|  | Family/ Desulfovibrionaceae |  |  | Multivariable Median | 0.048 | 0.097 | 0.625 | 1.05 (0.87-1.27) |
|  | Family/ Desulfovibrionaceae |  |  | Multivariable Egger | 0.066 | 0.067 | 0.326 | 1.07 (0.94-1.22) |
|  | Family/ Desulfovibrionaceae | Obesity class 3 | 20 | Multivariable IVW | -0.095 | 0.062 | 0.123 | 0.91 (0.81-1.03) |
|  | Family/ Desulfovibrionaceae |  |  | Multivariable Median | -0.102 | 0.089 | 0.251 | 0.90 (0.76-1.07) |
|  | Family/ Desulfovibrionaceae |  |  | Multivariable Egger | -0.093 | 0.062 | 0.130 | 0.91 (0.81-1.03) |
|  | Family/ Desulfovibrionaceae | Alcohol consumption | 17 | Multivariable IVW | 0.015 | 0.130 | 0.909 | 1.01 (0.79-1.31) |
|  | Family/ Desulfovibrionaceae |  |  | Multivariable Median | 0.000 | 0.165 | 1.000 | 1.00 (0.72-1.38) |
|  | Family/ Desulfovibrionaceae |  |  | Multivariable Egger | -0.008 | 0.135 | 0.954 | 0.99 (0.76-1.29) |
|  | Family/ Desulfovibrionaceae | Type 2 diabetes | 122 | Multivariable IVW | -0.096 | 0.065 | 0.140 | 0.91 (0.80-1.03) |
|  | Family/ Desulfovibrionaceae |  |  | Multivariable Median | -0.072 | 0.094 | 0.442 | 0.93 (0.77-1.12) |
|  | Family/ Desulfovibrionaceae |  |  | Multivariable Egger | -0.089 | 0.066 | 0.176 | 0.91 (0.80-1.04) |
|  | Family/ Desulfovibrionaceae | Ever smoked | 59 | Multivariable IVW | 0.264 | 0.577 | 0.647 | 1.30 (0.42-4.03) |
|  | Family/ Desulfovibrionaceae |  |  | Multivariable Median | 0.133 | 0.826 | 0.872 | 1.14 (0.23-5.77) |
|  | Family/ Desulfovibrionaceae |  |  | Multivariable Egger | 0.340 | 0.579 | 0.557 | 1.41 (0.45-4.38) |
|  | Genus / Senegalimassilia | High blood pressure | 331 | Multivariable IVW | 0.074 | 0.068 | 0.276 | 1.08 (0.94-1.23) |
|  | Genus / Senegalimassilia |  |  | Multivariable Median | 0.104 | 0.098 | 0.285 | 1.11 (0.92-1.34) |
|  | Genus / Senegalimassilia |  |  | Multivariable Egger | 0.084 | 0.068 | 0.218 | 1.09 (0.95-1.24) |
|  | Genus / Senegalimassilia | Obesity class 3 | 21 | Multivariable IVW | -0.080 | 0.062 | 0.193 | 0.92 (0.82-1.04) |
|  | Genus / Senegalimassilia |  |  | Multivariable Median | -0.119 | 0.088 | 0.176 | 0.89 (0.75-1.05) |
|  | Genus / Senegalimassilia |  |  | Multivariable Egger | -0.087 | 0.064 | 0.176 | 0.92 (0.81-1.04) |
|  | Genus / Senegalimassilia | Alcohol consumption | 17 | Multivariable IVW | -0.011 | 0.129 | 0.934 | 0.99 (0.77-1.27) |
|  | Genus / Senegalimassilia |  |  | Multivariable Median | -0.028 | 0.165 | 0.863 | 0.97 (0.70-1.34) |
|  | Genus / Senegalimassilia |  |  | Multivariable Egger | -0.023 | 0.138 | 0.867 | 0.98 (0.75-1.28) |
|  | Genus / Senegalimassilia | Type 2 diabetes | 117 | Multivariable IVW | -0.110 | 0.064 | 0.087 | 0.90 (0.79-1.02) |
|  | Genus / Senegalimassilia |  |  | Multivariable Median | -0.116 | 0.091 | 0.207 | 0.89 (0.74-1.07) |
|  | Genus / Senegalimassilia |  |  | Multivariable Egger | -0.110 | 0.064 | 0.087 | 0.90 (0.79-1.02) |
|  | Genus / Senegalimassilia | Ever smoked | 57 | Multivariable IVW | -0.415 | 0.600 | 0.489 | 0.66 (0.20-2.14) |
|  | Genus / Senegalimassilia |  |  | Multivariable Median | -0.834 | 0.859 | 0.331 | 0.43 (0.08-2.34) |
|  | Genus / Senegalimassilia |  |  | Multivariable Egger | -0.427 | 0.606 | 0.481 | 0.65 (0.20-2.14) |
|  | Genus / Ruminococcaceae_NK4A214_group | High blood pressure | 339 | Multivariable IVW | 0.063 | 0.067 | 0.352 | 1.06 (0.93-1.22) |
|  | Genus / Ruminococcaceae_NK4A215_group |  |  | Multivariable Median | 0.081 | 0.096 | 0.402 | 1.08 (0.90-1.31) |
|  | Genus / Ruminococcaceae_NK4A216_group |  |  | Multivariable Egger | 0.063 | 0.067 | 0.348 | 1.07 (0.93-1.22) |
|  | Genus / Ruminococcaceae_NK4A217_group | Obesity class 3 | 18 | Multivariable IVW | -0.094 | 0.062 | 0.128 | 0.91 (0.81-1.03) |
|  | Genus / Ruminococcaceae_NK4A218_group |  |  | Multivariable Median | -0.107 | 0.088 | 0.224 | 0.90 (0.76-1.07) |
|  | Genus / Ruminococcaceae_NK4A219_group |  |  | Multivariable Egger | -0.095 | 0.063 | 0.129 | 0.91 (0.80-1.03) |
|  | Genus / Ruminococcaceae_NK4A220_group | Alcohol consumption | 19 | Multivariable IVW | 0.054 | 0.131 | 0.680 | 1.06 (0.82-1.36) |
|  | Genus / Ruminococcaceae_NK4A221_group |  |  | Multivariable Median | -0.018 | 0.169 | 0.917 | 0.98 (0.71-1.37) |
|  | Genus / Ruminococcaceae_NK4A222_group |  |  | Multivariable Egger | 0.051 | 0.132 | 0.698 | 1.05 (0.81-1.36) |
|  | Genus / Ruminococcaceae_NK4A223_group | Type 2 diabetes | 125 | Multivariable IVW | -0.105 | 0.064 | 0.101 | 0.90 (0.79-1.02) |
|  | Genus / Ruminococcaceae_NK4A224_group |  |  | Multivariable Median | -0.102 | 0.092 | 0.263 | 0.90 (0.75-1.08) |
|  | Genus / Ruminococcaceae_NK4A225_group |  |  | Multivariable Egger | -0.105 | 0.064 | 0.099 | 0.90 (0.79-1.02) |
|  | Genus / Ruminococcaceae_NK4A226_group | Ever smoked | 62 | Multivariable IVW | -0.092 | 0.566 | 0.871 | 0.91 (0.30-2.77) |
|  | Genus / Ruminococcaceae_NK4A227_group |  |  | Multivariable Median | -0.507 | 0.813 | 0.532 | 0.60 (0.12-2.96) |
|  | Genus / Ruminococcaceae_NK4A228_group |  |  | Multivariable Egger | -0.091 | 0.588 | 0.877 | 0.91 (0.29-2.89) |
|  | Genus / Prevotella_7 | High blood pressure | 325 | Multivariable IVW | 0.069 | 0.067 | 0.301 | 1.07 (0.94-1.22) |
|  | Genus / Prevotella_7 |  |  | Multivariable Median | 0.049 | 0.097 | 0.613 | 1.05 (0.87-1.27) |
|  | Genus / Prevotella_7 |  |  | Multivariable Egger | 0.070 | 0.067 | 0.297 | 1.07 (0.94-1.22) |
|  | Genus / Prevotella_7 | Obesity class 3 | 16 | Multivariable IVW | -0.051 | 0.074 | 0.489 | 0.95 (0.82-1.10) |
|  | Genus / Prevotella_7 |  |  | Multivariable Median | -0.069 | 0.104 | 0.507 | 0.93 (0.76-1.14) |
|  | Genus / Prevotella_7 |  |  | Multivariable Egger | -0.061 | 0.091 | 0.506 | 0.94 (0.79-1.13) |
|  | Genus / Prevotella_7 | Alcohol consumption | 16 | Multivariable IVW | 0.008 | 0.119 | 0.949 | 1.01 (0.80-1.27) |
|  | Genus / Prevotella_7 |  |  | Multivariable Median | 0.005 | 0.155 | 0.975 | 1.00 (0.74-1.36) |
|  | Genus / Prevotella_7 |  |  | Multivariable Egger | 0.016 | 0.121 | 0.893 | 1.02 (0.80-1.29) |
|  | Genus / Prevotella_7 | Type 2 diabetes | 119 | Multivariable IVW | -0.100 | 0.065 | 0.125 | 0.90 (0.80-1.03) |
|  | Genus / Prevotella_7 |  |  | Multivariable Median | -0.083 | 0.094 | 0.376 | 0.92 (0.77-1.11) |
|  | Genus / Prevotella_7 |  |  | Multivariable Egger | -0.103 | 0.065 | 0.116 | 0.90 (0.79-1.03) |
|  | Genus / Prevotella_7 | Ever smoked | 55 | Multivariable IVW | -0.137 | 0.590 | 0.817 | 0.87 (0.27-2.77) |
|  | Genus / Prevotella_7 |  |  | Multivariable Median | -0.220 | 0.851 | 0.796 | 0.80 (0.15-4.25) |
|  | Genus / Prevotella_7 |  |  | Multivariable Egger | -0.124 | 0.590 | 0.834 | 0.88 (0.28-2.81) |
|  | Faecalibacterium | High blood pressure | 335 | Multivariable IVW | 0.070 | 0.068 | 0.306 | 1.07 (0.94-1.22) |
|  | Faecalibacterium |  |  | Multivariable Median | 0.053 | 0.098 | 0.590 | 1.05 (0.87-1.28) |
|  | Faecalibacterium |  |  | Multivariable Egger | 0.068 | 0.068 | 0.320 | 1.07 (0.94-1.22) |
|  | Faecalibacterium | Obesity class 3 | 16 | Multivariable IVW | -0.089 | 0.064 | 0.165 | 0.91 (0.81-1.04) |
|  | Faecalibacterium |  |  | Multivariable Median | -0.106 | 0.091 | 0.247 | 0.90 (0.75-1.08) |
|  | Faecalibacterium |  |  | Multivariable Egger | -0.085 | 0.091 | 0.352 | 0.92 (0.77-1.10) |
|  | Faecalibacterium | Alcohol consumption | 18 | Multivariable IVW | -0.018 | 0.129 | 0.889 | 0.98 (0.76-1.26) |
|  | Faecalibacterium |  |  | Multivariable Median | -0.033 | 0.163 | 0.841 | 0.97 (0.70-1.33) |
|  | Faecalibacterium |  |  | Multivariable Egger | 0.020 | 0.145 | 0.888 | 1.02 (0.77-1.36) |
|  | Faecalibacterium | Type 2 diabetes | 122 | Multivariable IVW | -0.097 | 0.064 | 0.126 | 0.91 (0.80-1.03) |
|  | Faecalibacterium |  |  | Multivariable Median | -0.093 | 0.093 | 0.314 | 0.91 (0.76-1.09) |
|  | Faecalibacterium |  |  | Multivariable Egger | -0.100 | 0.064 | 0.117 | 0.91 (0.80-1.03) |
|  | Faecalibacterium | Ever smoked | 60 | Multivariable IVW | -0.246 | 0.572 | 0.666 | 0.78 (0.25-2.40) |
|  | Faecalibacterium |  |  | Multivariable Median | -0.539 | 0.816 | 0.508 | 0.58 (0.12-2.88) |
|  | Faecalibacterium |  |  | Multivariable Egger | -0.261 | 0.573 | 0.648 | 0.77 (0.25-2.37) |

# Supplementary Table 13 Multivariable mendelian randomization analysis results between gut microbiota and thyroid nodules (adjusting five variables)

| **Taxa/gut microbiota** | **nSNP** | **Methods of multivariable MR** | **Beta** | **SE** | **p-value** | **OR** | **CI_lower** | **CI_upper** | **OR (95%CI)** |
| --- | --- | --- | --- | --- | --- | --- | --- | --- | --- |
| Class / Deltaproteobacteria | 304 | Multivariable IVW | -0.6207883 | 0.19328637 | 0.001319 | 0.537521 | 0.368016 | 0.785098 | 0.54 (0.37, 0.79) |
|  |  | Multivariable Median | -0.59333 | 0.239068 | 0.01307 | 0.552484 | 0.345797 | 0.882712 | 0.55 (0.35, 0.88) |
|  |  | Multivariable Egger | -0.64788 | 0.270437 | 0.01659 | 0.523154 | 0.307913 | 0.888853 | 0.52 (0.31, 0.89) |
| Order / Desulfovibrionales | 306 | Multivariable IVW | -0.60158564 | 0.191238321 | 0.001657 | 0.547942 | 0.37666 | 0.797113 | 0.55 (0.38, 0.80) |
|  |  | Multivariable Median | -0.4811 | 0.236374 | 0.041819 | 0.618103 | 0.388916 | 0.982351 | 0.62 (0.39, 0.98) |
|  |  | Multivariable Egger | -0.67762 | 0.267682 | 0.01136 | 0.507824 | 0.300509 | 0.858161 | 0.51 (0.30, 0.86) |
| Family / Lachnospiraceae | 310 | Multivariable IVW | 0.163487232 | 0.197232136 | 0.407157 | 1.17761 | 0.800045 | 1.733361 | 1.18 (0.80, 1.73) |
|  |  | Multivariable Median | 0.225398 | 0.23706 | 0.341703 | 1.252821 | 0.787226 | 1.993787 | 1.25 (0.79, 1.99) |
|  |  | Multivariable Egger | 0.551334 | 0.281744 | 0.050363 | 1.735567 | 0.999116 | 3.014857 | 1.74 (1.00, 3.01) |
| Family/ Desulfovibrionaceae | 306 | Multivariable IVW | -0.60012134 | 0.192318828 | 0.001806 | 0.548745 | 0.376414 | 0.799974 | 0.55 (0.38, 0.80) |
|  |  | Multivariable Median | -0.53362 | 0.237399 | 0.024592 | 0.586478 | 0.368276 | 0.933963 | 0.59 (0.37, 0.93) |
|  |  | Multivariable Egger | -0.64658 | 0.269156 | 0.016295 | 0.523834 | 0.309089 | 0.887777 | 0.52 (0.31, 0.89) |
| Genus / Senegalimassilia | 302 | Multivariable IVW | 0.20332319 | 0.135642616 | 0.133883 | 1.225468 | 0.939379 | 1.598687 | 1.23 (0.94, 1.60) |
|  |  | Multivariable Median | 0.386717 | 0.160571 | 0.016023 | 1.47214 | 1.074653 | 2.016647 | 1.47 (1.07, 2.02) |
|  |  | Multivariable Egger | 0.096785 | 0.208344 | 0.642258 | 1.101623 | 0.732297 | 1.657216 | 1.10 (0.73, 1.66) |
| Genus / Ruminococcaceae_NK4A214_group | 306 | Multivariable IVW | 0.193012979 | 0.777282001 | 0.946867 | 1.212899 | 0.264356 | 5.564937 | 1.21 (0.26, 5.56) |
|  |  | Multivariable Median | -0.27715 | 0.232509 | 0.233264 | 0.757941 | 0.480529 | 1.195504 | 0.76 (0.48, 1.20) |
|  |  | Multivariable Egger | -0.00104 | 0.280716 | 0.99704 | 0.998961 | 0.576233 | 1.731804 | 1.00 (0.58, 1.73) |
| Genus / Prevotella_7 | 299 | Multivariable IVW | -0.05459619 | 0.193012979 | 0.777282 | 0.946867 | 0.648624 | 1.382245 | 0.95 (0.65, 1.38) |
|  |  | Multivariable Median | -0.16777 | 0.11186 | 0.133664 | 0.845548 | 0.679081 | 1.052823 | 0.85 (0.68, 1.05) |
|  |  | Multivariable Egger | -0.1059 | 0.138048 | 0.442995 | 0.899515 | 0.686277 | 1.179009 | 0.90 (0.69, 1.18) |
| Genus / Faecalibacterium | 308 | Multivariable IVW | -0.10273679 | 0.19912887 | 0.605902 | 0.902364 | 0.610773 | 1.333165 | 0.90 (0.61, 1.33) |
|  |  | Multivariable Median | -0.16777 | 0.11186 | 0.133664 | 0.845548 | 0.679081 | 1.052823 | 0.85 (0.68, 1.05) |
|  |  | Multivariable Egger | -0.1059 | 0.138048 | 0.442995 | 0.899515 | 0.686277 | 1.179009 | 0.90 (0.69, 1.18) |

# Supplementary Table 14 Heterogeneity and Pleiotropy tests for multivariable MR analysis of gut microbiota and thyroid nodules

| **Outcome** | **Taxa/Gut microbiota** | **Adjusting variables** | **Multivariable Egger Intercept analysis** | | | **Cochran's Q test** | | | |
| --- | --- | --- | --- | --- | --- | --- | --- | --- | --- |
|  |  |  |  |  |  | **Multivariable IVW** | | **Multivariable MR-Egger** | |
|  |  |  | **Egger-intercept** | **SE** | ***P*-value** | **Q** | ***P*-value** | **Q** | ***P*-value** |
| Thyroid nodule | Class / Deltaproteobacteria | Hypertension | 0.000 | 0.004 | 0.903 | 350 | 0.284 | 350 | 0.271 |
|  |  | Obesity class 3 | 0.013 | 0.016 | 0.425 | 15 | 0.739 | 14 | 0.723 |
|  |  | Alcohol consumption | 0.015 | 0.022 | 0.491 | 9 | 0.937 | 9 | 0.928 |
|  |  | Type 2 diabetes | 0.003 | 0.006 | 0.591 | 113 | 0.687 | 113 | 0.670 |
|  |  | Ever smoked | 0.012 | 0.009 | 0.187 | 49 | 0.816 | 47 | 0.838 |
|  |  | All | 0.001 | 0.004 | 0.886 | 301 | 0.438 | 301 | 0.422 |
|  | Order / Desulfovibrionales | Hypertension | 0.003 | 0.004 | 0.411 | 349 | 0.281 | 349 | 0.281 |
|  |  | Obesity class 3 | 0.013 | 0.016 | 0.419 | 14 | 0.750 | 13 | 0.735 |
|  |  | Alcohol consumption | 0.016 | 0.022 | 0.449 | 8 | 0.952 | 7 | 0.948 |
|  |  | Type 2 diabetes | 0.005 | 0.006 | 0.425 | 112 | 0.697 | 111 | 0.689 |
|  |  | Ever smoked | 0.013 | 0.009 | 0.169 | 48 | 0.813 | 46 | 0.840 |
|  |  | All | 0.002 | 0.004 | 0.684 | 302 | 0.462 | 301 | 0.449 |
|  | Family / Lachnospiraceae | Hypertension | -0.005 | 0.004 | 0.211 | 358 | 0.180 | 356 | 0.186 |
|  |  | Obesity class 3 | 0.017 | 0.017 | 0.336 | 17 | 0.718 | 16 | 0.719 |
|  |  | Alcohol consumption | -0.014 | 0.026 | 0.599 | 13 | 0.787 | 13 | 0.749 |
|  |  | Type 2 diabetes | -0.009 | 0.007 | 0.215 | 110 | 0.646 | 108 | 0.660 |
|  |  | Ever smoked | -0.011 | 0.009 | 0.226 | 57 | 0.684 | 56 | 0.701 |
|  |  | All | -0.008 | 0.004 | 0.055 | 316 | 0.303 | 312 | 0.343 |
|  | Family/ Desulfovibrionaceae | Hypertension | 0.002 | 0.004 | 0.517 | 349 | 0.285 | 349 | 0.277 |
|  |  | Obesity class 3 | 0.013 | 0.016 | 0.426 | 15 | 0.687 | 14 | 0.667 |
|  |  | Alcohol consumption | 0.013 | 0.022 | 0.548 | 7 | 0.954 | 7 | 0.944 |
|  |  | Type 2 diabetes | 0.005 | 0.006 | 0.416 | 112 | 0.698 | 111 | 0.691 |
|  |  | Ever smoked | 0.012 | 0.009 | 0.175 | 48 | 0.800 | 46 | 0.826 |
|  |  | All | 0.001 | 0.004 | 0.805 | 302 | 0.453 | 302 | 0.437 |
|  | Genus / Senegalimassilia | Hypertension | 0.001 | 0.004 | 0.814 | 344 | 0.263 | 344 | 0.251 |
|  |  | Obesity class 3 | -0.026 | 0.018 | 0.149 | 14 | 0.452 | 12 | 0.536 |
|  |  | Alcohol consumption | -0.006 | 0.027 | 0.830 | 8 | 0.635 | 8 | 0.545 |
|  |  | Type 2 diabetes | 0.005 | 0.007 | 0.483 | 108 | 0.721 | 107 | 0.709 |
|  |  | Ever smoked | 0.008 | 0.010 | 0.436 | 51 | 0.496 | 51 | 0.480 |
|  |  | All | 0.003 | 0.004 | 0.500 | 304 | 0.359 | 304 | 0.350 |
|  | Genus / Ruminococcaceae_NK4A214_group | Hypertension | -0.005 | 0.004 | 0.250 | 356 | 0.233 | 354 | 0.237 |
|  |  | Obesity class 3 | 0.002 | 0.016 | 0.880 | 15 | 0.503 | 15 | 0.432 |
|  |  | Alcohol consumption | -0.003 | 0.025 | 0.904 | 15 | 0.577 | 15 | 0.507 |
|  |  | Type 2 diabetes | 0.005 | 0.006 | 0.419 | 117 | 0.632 | 116 | 0.624 |
|  |  | Ever smoked | 0.000 | 0.009 | 0.997 | 61 | 0.455 | 61 | 0.419 |
|  |  | All | -0.001 | 0.004 | 0.792 | 317 | 0.236 | 317 | 0.225 |
|  | Genus / Prevotella_7 | Hypertension | 0.005 | 0.004 | 0.214 | 325 | 0.454 | 324 | 0.463 |
|  |  | Obesity class 3 | 0.004 | 0.022 | 0.862 | 11 | 0.660 | 11 | 0.585 |
|  |  | Alcohol consumption | 0.012 | 0.026 | 0.639 | 5 | 0.980 | 5 | 0.980 |
|  |  | Type 2 diabetes | 0.005 | 0.006 | 0.392 | 106 | 0.749 | 106 | 0.744 |
|  |  | Ever smoked | -0.012 | 0.009 | 0.177 | 46 | 0.751 | 44 | 0.780 |
|  |  | All | 0.002 | 0.004 | 0.596 | 300 | 0.375 | 300 | 0.364 |
|  | Genus / Faecalibacterium | Hypertension | 0.003 | 0.004 | 0.524 | 354 | 0.209 | 353 | 0.202 |
|  |  | Obesity class 3 | -0.002 | 0.024 | 0.949 | 13 | 0.548 | 13 | 0.469 |
|  |  | Alcohol consumption | 0.013 | 0.023 | 0.565 | 6 | 0.983 | 6 | 0.978 |
|  |  | Type 2 diabetes | 0.014 | 0.006 | 0.025 | 113 | 0.659 | 108 | 0.753 |
|  |  | Ever smoked | 0.004 | 0.008 | 0.672 | 48 | 0.812 | 48 | 0.790 |
|  |  | All | 0.008 | 0.004 | 0.064 | 319 | 0.234 | 316 | 0.266 |
